# A haplotype map of allohexaploid wheat reveals distinct patterns of selection on homoeologous genomes

**DOI:** 10.1186/s13059-015-0606-4

**Published:** 2015-02-26

**Authors:** Katherine W Jordan, Shichen Wang, Yanni Lun, Laura-Jayne Gardiner, Ron MacLachlan, Pierre Hucl, Krysta Wiebe, Debbie Wong, Kerrie L Forrest, IWGS Consortium, Andrew G Sharpe, Christine HD Sidebottom, Neil Hall, Christopher Toomajian, Timothy Close, Jorge Dubcovsky, Alina Akhunova, Luther Talbert, Urmil K Bansal, Harbans S Bariana, Matthew J Hayden, Curtis Pozniak, Jeffrey A Jeddeloh, Anthony Hall, Eduard Akhunov

**Affiliations:** Department Plant Pathology, Kansas State University, Manhattan, KS 66506 USA; Integrated Genomics Facility, Kansas State University, Manhattan, KS 66506 USA; Institute of Integrative Biology, University of Liverpool, Liverpool, L69 7ZB UK; Department Plant Sciences, University of Saskatchewan, Saskatoon, SK S7N 5A8 Canada; Department Environment and Primary Industries, Bundoora, VIC 3083 Australia; National Research Council Canada, 110 Gymnasium Place, Saskatoon, SK S7N 0 W9 Canada; Department Botany & Plant Sciences, University of California, Riverside, CA 92521 USA; Department Plant Sciences, University of California, Davis, CA 95616 USA; Howard Hughes Medical Institute, Chevy Chase, MD 20815 USA; Department Plant Sciences & Plant Pathology, Montana State University, Bozeman, MT 59717 USA; Plant Breeding Institute-Cobbitty, The University of Sydney, PMB4011, Narellan, NSW 2567 Australia; Roche NimbleGen, Inc, Madison, WI 53719 USA

## Abstract

**Background:**

Bread wheat is an allopolyploid species with a large, highly repetitive genome. To investigate the impact of selection on variants distributed among homoeologous wheat genomes and to build a foundation for understanding genotype-phenotype relationships, we performed population-scale re-sequencing of a diverse panel of wheat lines.

**Results:**

A sample of 62 diverse lines was re-sequenced using the whole exome capture and genotyping-by-sequencing approaches. We describe the allele frequency, functional significance, and chromosomal distribution of 1.57 million single nucleotide polymorphisms and 161,719 small indels. Our results suggest that duplicated homoeologous genes are under purifying selection. We find contrasting patterns of variation and inter-variant associations among wheat genomes; this, in addition to demographic factors, could be explained by differences in the effect of directional selection on duplicated homoeologs. Only a small fraction of the homoeologous regions harboring selected variants overlapped among the wheat genomes in any given wheat line. These selected regions are enriched for loci associated with agronomic traits detected in genome-wide association studies.

**Conclusions:**

Evidence suggests that directional selection in allopolyploids rarely acted on multiple parallel advantageous mutations across homoeologous regions, likely indicating that a fitness benefit could be obtained by a mutation at any one of the homoeologs. Additional advantageous variants in other homoelogs probably either contributed little benefit, or were unavailable in populations subjected to directional selection. We hypothesize that allopolyploidy may have increased the likelihood of beneficial allele recovery by broadening the set of possible selection targets.

**Electronic supplementary material:**

The online version of this article (doi:10.1186/s13059-015-0606-4) contains supplementary material, which is available to authorized users.

## Background

**Figure 1:**
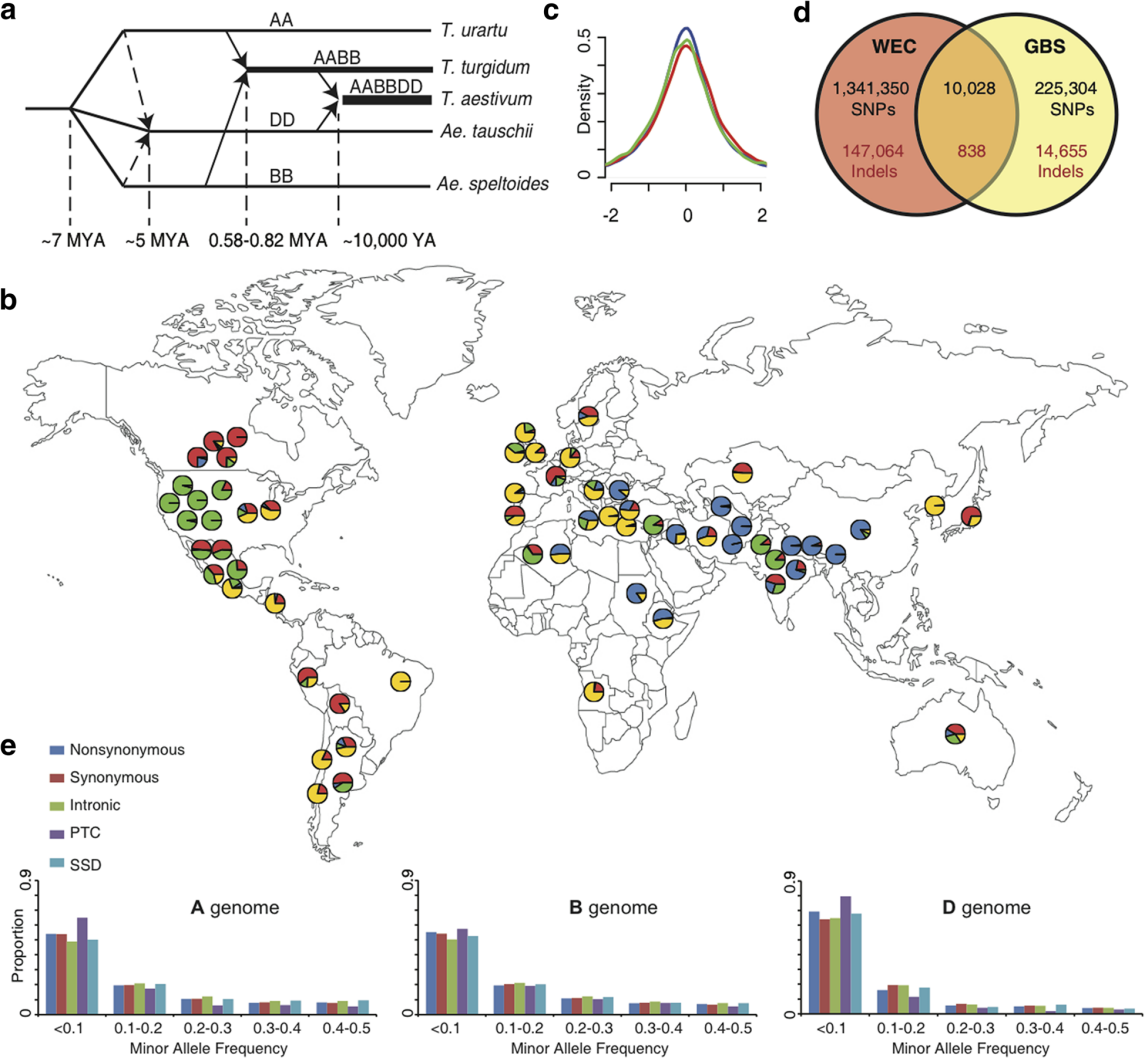
**Summary of re-sequencing panel. (a)** Evolution of the hexaploid wheat genome. The tetraploid wheat *T. turgidum* (AABB) originated by the hybridization of *T. urartu* with the close unidentified relative of *Ae. speltoides* occurred about 0.58 to 0.82 million years ago according to the genome-wide divergence time estimate [10]. The origin of hexaploid wheat occurred about 10,000 years ago [11] by the hybridization of *T. turgidum* with *Ae. tauschii* (DD) [12]. Marcussen et al. [10] suggested that *Ae. tauschii* might have originated by homoploid hybrid speciation (shown by dashed arrows). **(b)** Geographic distribution of 62 accessions of wheat accessions. Pie charts indicate the proportion of genetic ancestry for K = 4 inferred using Structure. **(c)** Efficiency of homoeologous gene capture. The depth of read coverage was extracted for each of the three copies of 47,739 homoeologous gene sets. The histogram of the log_2_ transformed ratio of read coverage between A and B (red), A and D (blue), and B and D (green) genomes was plotted. Each plot shows a normal distribution with the overall mean at 0. **(d)** Overlap between the SNP and indel datasets generated by WEC and GBS. **(e)** Minor allele frequency of different functional classes of SNPs as a proportion of total SNPs within each genome and class. PTC: premature termination codons; SSD: splice-site disruptions.

Wheat genomic variation is shaped by the interplay of multiple factors including two recent polyploidization events [1-3] (Figure 1a), domestication [4], spread from the sites of origin to new geographic regions, gene flow from the populations of wild and domesticated ancestors [5], and post-domestication selection aimed at developing high-yielding locally adapted varieties. The eco-geographic habitats to which wheat is adapted span diverse environments ranging from low humidity regions in Nigeria, and the northern regions of Russia and Norway to the high-humidity regions of South America and Bangladesh [6]. It has been suggested that this broad adaptability likely results from the genetic diversity captured from the natural populations of its tetraploid ancestors [5,7] combined with a high rate of evolutionary changes in the wheat genome (particularly insertions and deletions), which are tolerated by its polyploid nature [8,9].

A detailed description of DNA sequence variation across the genome is a prerequisite for the systematic analysis of variants underlying trait variation in wheat and critical for understanding the role of various evolutionary factors in shaping genome diversity. Recently, low to medium density genotyping arrays were used to characterize SNP variation and linkage disequilibrium (LD) in wheat populations [13,14] and identify variants associated with phenotypic traits [15,16]. However, despite being a useful genotyping tool, these arrays are incapable of capturing the entire spectrum of DNA sequence variation and allele frequencies in wheat populations, and providing unbiased information that may help directly identify causal variants affecting phenotypes. Achieving this goal requires obtaining sequence data on a genome-scale from a diverse population of lines. To date, this has only been performed on limited samples of wheat lines used to discover SNPs in the parental lines of mapping populations [17], or for SNP-based array design [18].

Genome sequencing of populations of individuals has been undertaken in a number of species including humans, *Arabidopsis*, and several crops [19-23] and helped to detect alleles contributing to phenotypic variation and adaptation. Despite recent advances in next-generation sequencing (NGS), performing similar analyses of genomic variation in wheat is substantially complicated by allopolyploidy and large genome size (approximately 17 Gb). However, sequencing of DNA samples subjected to complexity reduction by exome capture [18,24] and genotyping by sequencing [25,26] was shown to be an effective strategy to analyze the complex genomes. In addition, the recent release of the chromosome-specific wheat genome assemblies [27,28] can now help to alleviate the problems associated with variant calling in the allopolyploid genome, and allow us to describe the chromosomal distribution of variants and their potential effect on gene function.

Here we used the newly developed genome assembly of the cultivar Chinese Spring [27] based on flow-sorted chromosome survey sequence (CSS) contigs to create a diversity map of allopolyploid bread wheat. The data were generated by re-sequencing 62 diverse wheat lines using whole exome capture (WEC) and genotyping by sequencing (GBS) approaches. The panel of wheat lines was selected to capture the genetic diversity of the major global wheat growing regions and included landraces and cultivars (Figure 1b; Table S1 and Figure S1 in Additional file 1). We used the obtained data to describe the effect of genetic variation on gene function, gain insights into the effect of selection on duplicated genes, and explore the LD landscape in each of the three wheat sub-genomes to better understand the role of selection in shaping the genetic diversity of wheat.

## Results and discussion

### Re-sequencing the allopolyploid wheat genome

The WEC assay probes were designed with 107 Mb of non-redundant low-copy genic regions [29] targeting nearly 321 Mb of sequence in all three wheat genomes (Figure S2 in Additional file 1). The capture probes covered 78% of the 124,201 high-confidence protein-coding genes (at the 95% similarity threshold) in the CSS contigs [27]. The GBS approach generated sequence data primarily outside the genic regions. We produced roughly 4.7 billion paired-end reads (4.5 billion WEC reads and 0.2 billion GBS reads), and 62% of WEC reads and 51% of GBS reads uniquely mapped to the CSS contigs of the individual chromosomes (Figure S3 and Tables S2, S3 in Additional file 1), using alignment parameters optimized to separate reads from the different wheat genomes (Figures S4 and S5 in Additional file 1). In the WEC dataset, similar relative read coverage across homoeologous targets indicated that non-redundant capture probes are capable of recovering sequences from the different genomes with equal efficiency (Figure 1c, Table S4 in Additional file 1).

Variant calling was performed in the regions of the wheat genome covered by reads in >46 lines (>75%). We identified 1.57 million single nucleotide polymorphisms (SNPs) and 161,719 small insertions-deletions (indels) distributed across all 21 chromosomes, producing an average density of 1 variant every 175 bp (Figure 1d; Figure S6, Tables S5 and S6 in Additional file 1). Consistent with the previous estimates of genetic diversity [30], the A (649,522) and B (791,971) genomes contained about 2.5 times more variants than the D genome (286,880).

The overall genotype error rates for SNPs and indels assessed by comparing genotype calls generated for cultivar Chinese Spring with the CSS contigs of the same cultivar were 1.1% and 1.5%, respectively (for details see Materials and Methods). The error rate for rare SNPs and indels covered by >10 reads was 4.6% and 3.4%, respectively (Figure S7 in Additional file 1). The majority (77%) of variants in the GBS dataset were found in intergenic regions (Table S5 in Additional file 1), and only 4.3% of the variants overlapped with the WEC dataset (Figure 1d). This finding is supported by the *in silico* PstI digest of the CSS contigs, which showed that the regions targeted by the GBS cover only 6.8% of the regions targeted by the WEC.

### Impact of purifying selection on genetic variation in the polyploid genome

One of the predicted consequences of whole genome duplication is functional redundancy that can result in the relaxation of purifying selection acting on duplicated copies of genes, thereby increasing the rate of accumulation of functional mutations. Previous studies suggested that polyploidy can result in the accelerated accumulation of premature termination codons in coding sequences [9] or an excess of non-synonymous changes in the polyploid lineage compared to the lineages of its diploid ancestors [27]. However, the ability of polyploid wheat to tolerate aneuploidy or large-scale deletions suggests that the duplicated homoeologs can be functional. It is not known whether this functionality is maintained by purifying selection or, as a consequence of redundancy and selection relaxation, subject to decay through the mutation process.

The wheat genome contains a number of variants that are predicted to impact gene function. We found 6,944 indels that could have a negative impact on gene function resulting from a predicted reading frame shift (Table S7 in Additional file 1). The proportion of frame-shift indels, relative to in-frame indels, in the coding regions of the wheat genome (67%) was higher than that reported (57%) in the human genome [19]. There were twice as many indels with a length of a multiple of 3 located within the coding regions than within the introns or untranslated regions (Figure S8 in Additional file 1), indicative of purifying selection maintaining reading frame within the coding regions.

In coding sequences we identified 83,622 non-synonymous and 76,361 synonymous SNPs (Table S5 in Additional file 1). Based on high-confidence gene models in the CSS contigs, we determined that only 1,600 and 1,583 SNPs are predicted to produce premature termination codons (PTCs) and splice-site disruptions (SSDs), respectively, with a two- to three-fold lower incidence of functional mutations in the D genome than in the A and B genomes (Table S6 in Additional file 1). Out of the 6,230 genes that have homoeologous copies in the wheat genome and harbor coding sequence-disrupting mutations including frame-shift indels and PTCs, 4,870 (78%) have at least one intact homoeologous copy suggesting that the deleterious effects of these variants, if any, could be compensated. The compensatory potential of the duplicated homoeologous genes is consistent with a higher density of chemically induced mutations (five- to eight-fold) that can be achieved in wheat compared to other diploid plant species [31]. However, in spite of the presence of intact functional homoeologous copies of genes, we found a reduced number of non-synonymous, PTC, and SDS variants with a high derived allele frequency in the population (Figure 1e; Figure S9 in Additional file 1). This depletion of functional variants at higher allele frequencies is consistent with purifying selection acting against functional mutations, and suggests that the effect of purifying selection is not completely diminished by whole genome duplication. There are two plausible explanations for the retention of functional gene copies in young polyploids. First, selection acts on gene function that was partitioned among the homoeologs after the whole genome duplication. The functional partitioning is consistent with the studies of natural and artificial polyploids of wheat and other plants, often showing the tissue- and/or development-specific expression of homoeologs [32-35]. Alternatively, selection favors functional homoeologs to maintain the optimal stoichiometric ratios of gene products in macromolecular complexes, or in multistep regulatory cascades, which was proposed in the gene balance hypothesis [36].

The fraction of non-synonymous changes varied significantly among different protein families (Table S8 in Additional file 2) with the majority of PFAM domains involved in basic cellular functions showing a reduced proportion of non-synonymous changes compared to the genome-wide value, indicative of strong purifying selection. We detected a significant enrichment (*χ*2 test *P* value <10^-4^, Table S8 in Additional file 2) for non-synonymous changes in the LRR and NB-ARC domains of disease resistance genes. The enrichment for major effect SNPs in these genes appears to be common for plant genomes and was also found in *Arabidopsis* [37] and peanut [38]. These observations are consistent with the hypothesis of an ‘arms race’ between the evolving populations of a pathogen and a plant defense system that results in fast evolution of genes with new disease-resistance specificities [39].

### Global patterns of genetic variation

**Figure 2:**
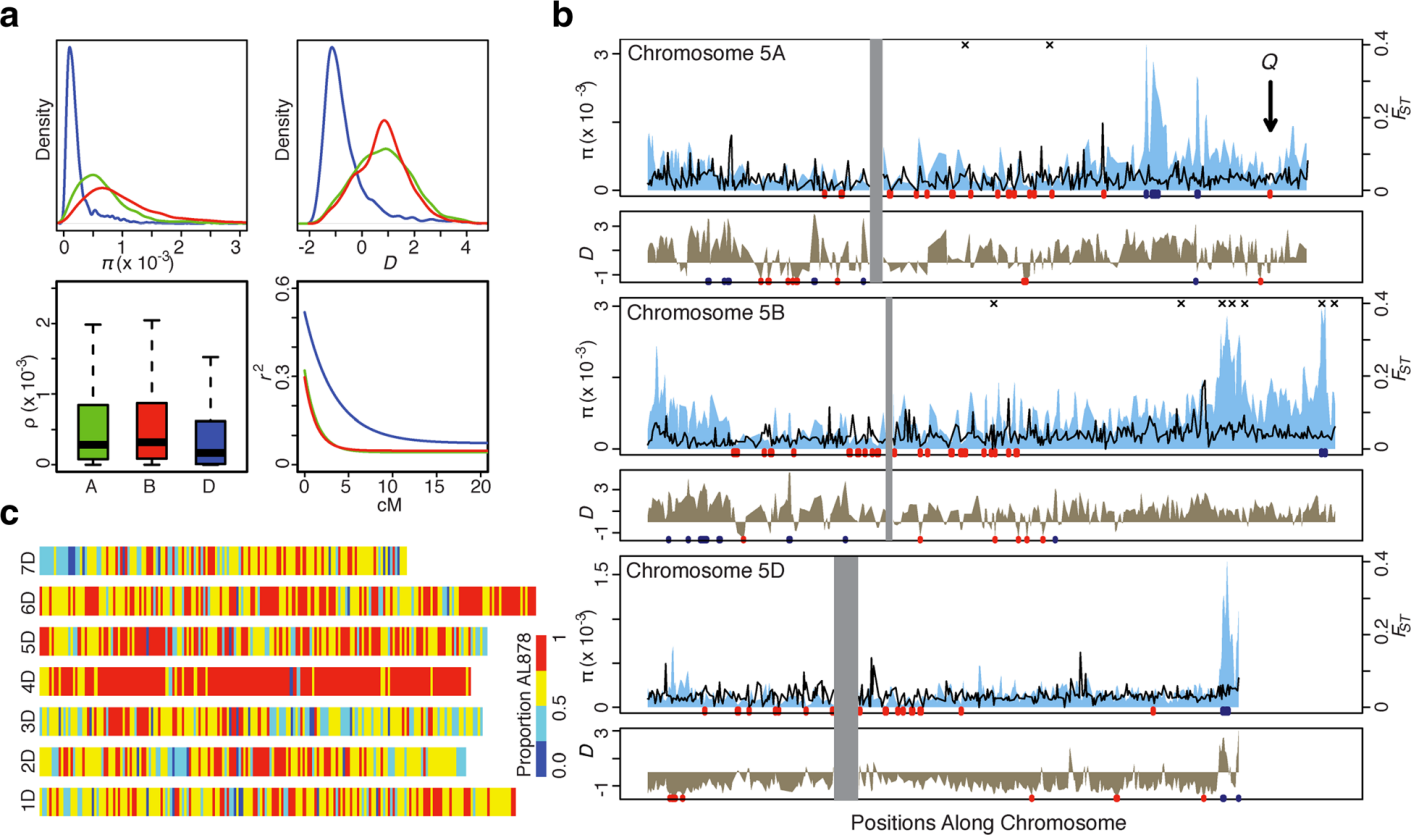
**Distribution of genetic diversity, allele frequency, and recombination across the wheat genome. (a)** Distribution of genetic diversity in the A (green), B (red), and D (blue) genomes: (π, top left), Tajima’s measure of site frequency spectrum (D, top right), historic recombination (ρ, bottom left), and LD (bottom right). **(b)** Distribution of nucleotide diversity π (shaded polygon), F_ST_ between cultivars and landraces (solid black line), and site frequency spectrum (D) along chromosomes 5A (top panel), 5B (middle panel) and 5D (bottom panel). Gray shaded boxes represent the approximate location of the centromere. Rug plots represent lower (red) and upper (blue) 2.5% tails of test statistic distribution. Black X above the plot represents upper 2.5% tail of ρ statistic. The location of domestication (*Q*) gene is shown by arrow. **(c)**. Distribution of alleles of the AL8/78 genotype of *Ae. tauschii* along the chromosomes of the D genome in the 26 wheat landraces. The average frequency of AL8/78 alleles was calculated in a 3 Mb sliding window. The color scale shows the proportion of the AL8/78 alleles in each window (red - highest, blue - lowest).

The global patterns of genomic variation and distribution of inter-variant associations are impacted by historic selection and demographic events, and by variation in recombination rate [40]. We found a non-random variant distribution along the chromosomes with reduced variation near the centromeres and elevated variation at the telomeres (Figure 2a and b; Figures S10-15 in Additional file 1), which is consistent with previous studies [28,30]. This pattern is similar to what was reported for maize and humans [19,41], but differs from *Arabidopsis* [37], where regions of high polymorphism were located near the centromeres. Our data also showed reduced diversity and an excess of rare alleles in the D genome when compared to the A and B genomes (Figure 2a; Table S9 in Additional file 1) [30]. These trends are consistent with the hypothesis that the limited number of ancestral genotypes of the D genome contributed to the origin of hexaploid wheat [42]. An elevated level of diversity in the A and B genomes, which otherwise would be expected to show the same levels of diversity as the D genome, could be attributed to the influx of allelic variation from the sympatric populations of wild tetraploid relatives [7,43].

Differentiation between landraces and cultivars (*F*_ST_) varied along chromosomes with lower values found near the telomeres (Figure 2b; Figures S10-15 in Additional file 1). Long stretches of elevated *F*_ST_ were found along chromosome 4A and the short arm of chromosome 7B, two of the most structurally re-arranged chromosomes in the wheat genome [44] (Figure S16 in Additional file 1). Since these structural re-arrangements are fixed in wheat and, therefore, unlikely to affect gene flow between populations resulting in high *F*_ST_, the overlap of differentiated genomic regions with those showing the signal of positive selection (Table S10 in Additional file 3) suggests that the detected differentiation could be associated with improvement selection.

Identification of genomic regions showing positive Tajima’s *D* (excess of common alleles) and elevated diversity in the D genome (Figure 2b; Figures S10-15 in Additional file 1) is indicative of the presence of highly diverged haplotypes in our sample most likely resulting from introgressions. To test this possibility, we compared the D genome haplotypes of 26 landraces not affected by modern breeding with the sequence of *Ae. tauschii* accession AL8/78 [45], which is considered the most closely related to the wheat D genome [46]. The high proportion of AL8/78 alleles (74%) and their distribution along the wheat chromosomes confirms the ancestry of the D genome (Figure 2c) [42]. However, the fine-scale haplotypic structure also reveals regions carrying highly divergent haplotypes (Figure 2c) suggestive of significant levels of introgression from either the diverged *Ae. tauschii* lines or independently originated hexaploid wheat lineages founded by diverged *Ae. tauschii* genotypes. The preferential localization of introgressions in the high-recombining regions of the chromosomes indicates that gene flow between the different D genome lineages was uneven along the chromosomes.

Using re-sequencing data, we now have the possibility to assess historic recombination rate (parameter ρ) [47], which is the product of meiotic recombination rate variation and effective population size. The median estimate of ρ in the D genome (ρ = 1.7 × 10^-4^/kb) was lower than in the A (ρ = 2.9 × 10^-4^/kb) and B (ρ = 3.2 × 10^-4^/kb) genomes (Figure 2a; Table S9 in Additional file 1). Consistent with the observations made in *Arabidopsis*, maize, and humans [40,41,48], we detected a positive correlation in the A (Spearman *r*_*sp*_^2^ = 0.23, *P* <10^-9^) and B (*r*_*sp*_^2^ = 0.21, *P* <10^-12^) genomes between the meiotic (R = genetic distance in cM / physical distance in Mb) and historic recombination rates suggesting stability of chromosomal recombination rates over time. However, no significant correlation was found between R and ρ in the D genome. This fact is most likely explained by the polyploidy-associated population bottleneck, which can impact the estimates of historic recombination [49] and also can result in reduced ρ in the D genome compared to that in the A and B genomes.

The historic recombination and diversity in wheat showed a positive correlation with relative distance from the centromere (*r*_*sp*_^*2*^ = 0.15, *P* <10^-4^), a trend previously reported in maize, and humans [41,48]. These relationships likely explain most of the inter-genomic diversity correlation along the homoeologous chromosomes (Table S11 in Additional file 1). Except for a few cases, we found low inter-genomic correlation of the window-based Tajima’s D and F_ST_ estimates along the duplicated genomic regions of homoeologous chromosomes. This outcome is most likely a consequence of the sensitivity of these summary statistics to historic demographic and selection events that likely had different impacts on each wheat genome (Table S11 in Additional file 1).

### Genotype imputation and GWAS

In genome-wide association studies (GWAS), marker density affects the probability of finding variants in linkage disequilibrium (LD) with a causal variant. A SNP-hiding test [40] showed that the probability of identifying high-LD SNPs (*r*^2^ > 0.8) in our population within a 2-kb window was 70% to 71% for all three genomes. Consistent with the observed LD levels in the wheat genomes (Figure 2a), for SNPs located from 2 kb to 4 kb apart, the probability of finding high-LD SNPs in the D genome (56%) was higher than in the A and B genomes (48% to 49%).

Diversity maps have proven to be a powerful tool for imputing genotypes [19,20] allowing for an increase in marker density and the precision of trait mapping. Using a common set of SNP markers shared between the WEC data and the 90 K SNP assay [14] currently used by the community to genotype large numbers of wheat accessions, we tested the utility of our data for genotype imputation.

**Figure 3:**
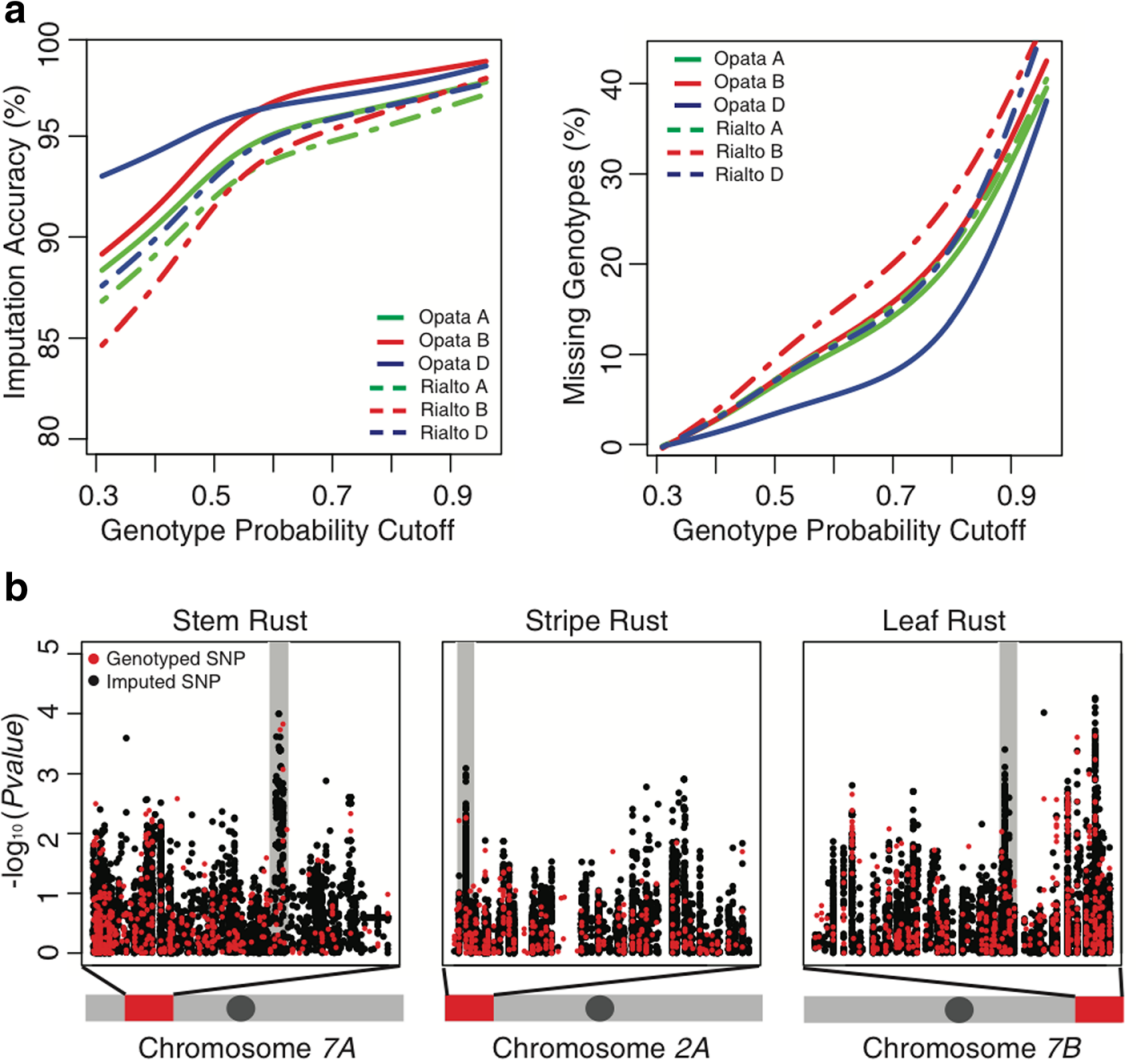
**Genotype imputation. (a)** Relationship between the accuracy of genotype imputation and the percentage of missing data, which is estimated after removing genotypes over a range of genotype calling probability thresholds. Imputation in Opata (solid lines) and Rialto (dashed lines) cultivars was performed using the reference panel of 60 lines (Opata and Rialto cultivars were excluded) genotyped using the 90 K iSelect assay. **(b)** Genotype imputation at disease resistance loci. Two GWAS regions overlapped with the positions of the previously mapped *Lr37/Yr17/Sr38* (middle panel) and *Lr68* (right panel) disease resistance loci; the markers associated with these loci showed highest similarity to CSS contigs 2AS-5264433 and 7BL-6748067, respectively. SNP sites directly genotyped using the 90 K SNP array are shown as red dots; imputed SNPs are shown as black dots.

First, we sequentially selected each cultivar from our panel of 62 lines and, after ‘hiding’ all SNP sites besides those overlapping with the public 90 K SNP array [14], we used the WEC SNP data in the remaining 61 lines to predict the ‘hidden’ variants. Depending on the selected wheat line, using a genotype calling probability cutoff of 0.6, the accuracy of genotype predictions assessed by comparing with the observed data was in the range of 93% to 97% (Figure 3a; Table S12 in Additional file 4). This genotype probability cutoff value resulted in the removal of 5% to 15% of the data (Figure 3a), and allowed imputation of up to 549,918 SNPs. The accuracy of SNP imputation varied among the wheat genomes reflecting the inter-genomic differences in the extent of LD (Figure 2a). For example, the highest imputation accuracy was achieved for the D genome, which also showed the highest levels of inter-variant LD.

Second, we used our reference panel of 62 accessions to impute DNA polymorphisms in a GWAS. A panel of 678 diverse wheat landraces phenotyped for resistance to three rust diseases (Tables S13-S16 in Additional files 5, 6, 7, and 8) [15] was tested for marker-trait associations using genotyping data generated with the wheat 90 K SNP array. In this panel we were able to impute 344,544 SNPs, of which 210,017 SNPs with a MAF above 3% and the proportion of missing data less than 80% were used for GWAS. Three selected marker-trait associations were validated by mapping in the populations of recombinant inbred lines; two of these associations correspond to disease resistance loci *Lr67* and *Yr51*, and one represents an uncharacterized locus (Figure 3b) associated with stem rust resistance (Tables S13-S16 in Additional files 5, 6, 7, and 8). Two additional regions from our GWAS were shown to overlap with the positions of the previously mapped *Lr37/Yr17/Sr38* and *Lr68* disease resistance loci (Figure 3b). The markers associated with these loci showed highest similarity to CSS contigs 2AS-5264433 and 7BL-6748067, respectively [27,50]. Overall, comparison of marker-trait associations at non-imputed and imputed sites shows that imputed SNPs not only increase marker density but in most cases perform similar to or better than the SNPs directly genotyped using the 90 K assay (Figure 3b; Figure S17 in Additional file 1). These results demonstrate the value of having a more complete ascertainment of DNA polymorphisms for GWAS that is achieved utilizing the high-density SNP variation data developed from 62 lines and the public 90 K SNP genotyping array [14].

### Signatures of selection in the polyploid genome

During the development of adapted lines, selection imposed by humans favored alleles controlling traits valuable for agriculture. When selection increases the frequency of beneficial alleles in a population, it impacts the standing variation of surrounding genomic regions resulting in reduced diversity, extended linkage disequilibrium, or strong inter-population allele frequency differentiation [51]. To detect these local patterns of variation, also referred to as ‘selective sweeps’, we investigated the patterns of genetic variation along the chromosomes and used two complementary approaches based on cross-population composite likelihood ratio (XP-CLR) [52] (Figure S18 in Additional file 1) and pair-wise haplotype sharing (PHS) [53] tests (Tables S17-S19 in Additional files 1, 9, and 10).

The reduced diversity observed near the wheat domestication genes *Q* and *Tg* was consistent with selection at the early stages of domestication (Figure 2b; Figure S11 in Additional file 1) [54,55]. For example, the CSS contig 1750512_5AL harboring the *Q* gene harbors only one intronic SNP at position 1249. Regions harboring genes known to be associated with local adaptation (*Ppd*, *Vrn*, *Rht*) also showed selection scan test statistic scores approaching the extremes (Table S20 in Additional file 1). To further validate that our selection scans detect genomic regions associated with candidate loci controlling agronomic traits targeted by humans during the development of improved varieties, we compared selective sweeps with the marker-trait associations identified in mapping studies. We found 474 previously published associations that fell within the target regions (Table S21 in Additional file 11). These markers showed association with major domestication and agronomic traits including spike length, rachis fragility, and compactness [56,57], heading date and flowering time [16,58], grain shape and yield characteristics [59,60], and nitrogen use efficiency [61]. The selective sweep regions also included markers associated with resistance to stripe rust [50], bacterial leaf streak, and spot blotch [62,63]. The regions detected in the PHS scan overlapped with 459 marker-trait associations. Far fewer previously associated markers were located within the outliers of the F_ST_, XP-CLR, and diversity scans.

Non-synonymous, that is, likely functional, variants were significantly enriched (*χ*2 test, *P* value <5.4 × 10^-11^) in the extreme tails of the selection scans compared to synonymous variants. Regions identified by multiple selection scans were not common (Table S22 in Additional file 1), and the regions where all three scans overlapped contained no genes with annotations. Among the overlapping regions detected using two different methods, one of the most common classes of genes were disease resistance genes (Table S10 in Additional file 3). Consistently, the NB-ARC and LRR encoding domains of genes involved in disease resistance pathways [64] were significantly over-represented in the extreme tail of the PHS scan, (*χ*2 test; FDR <10^-5^ and <10^-2^, respectively) (Table S23 in Additional file 1) suggesting that some targets of selection can be associated with selection for disease resistance. To confirm this hypothesis, we performed GWAS of resistance to leaf, stem, and stripe rust (Figure 3b, Materials and methods) and tested for enrichment of marker-trait associations in the extreme tail of the selection scan. In the upper 2.5% tail of the PHS scan, we found three-, four-, and five-fold enrichment of SNPs (*χ*2 test, *P* <6.5 × 10^-6^) associated with resistance to stem, leaf, and stripe rust, respectively, without the concomitant enrichment in the XP-CLR scan. Since the PHS test preferentially detects on-going selection events that have not reached fixation in a population [53], our results suggest that multiple disease resistance genes undergo selection across wheat populations, which is likely associated with the spatial and temporal variation in pathogen populations, and consistent with the ‘arms race’ hypothesis [39].

Among other candidates of selection is the WRKY transcription factor (Ta1dsLoc014113), identified by both selection scans and located on the short arm of chromosome 1D; its expression was shown to be associated with resistance to a fungal pathogen causing powdery mildew in wheat [65]. Glutathione-S-transferase encoding genes (Ta7dsLoc015003, Ta7bsLoc009692), that play an important role in drought response by reducing the toxicity of reactive oxygen species in wheat and other plants [66,67], were identified by the PHS and *F*_ST_ scans on chromosomes 7B and 7D (Table S10 in Additional file 3).

The development of the chromosome-specific wheat genome assemblies and population-scale whole exome re-sequencing data now provides the unique opportunity to investigate the impact of selection on duplicated copies of homoeologous genes. We have used the ordered sets of homoeologous genes to establish the syntenic relationships between the selection targets on different genomes. Inter-genomic comparison of selection targets associated with transition from landraces to cultivars revealed that only a single syntenic region shared the signature of selection between the B and D genomes (Table S24 in Additional file 1). This region on the long arm of chromosome 1 shared two annotated genes encoding cellulose synthase (Ta1dlLoc001027, Ta1blLoc007155, Ta1blLoc025394), which plays a central role in cellulose biosynthesis, and trehalase (TH) (Ta1dlLoc015131, Ta1blLoc007983). The latter gene contains a PFAM domain that was significantly enriched in the tail of the XP-CLR scan (Table S23 in Additional file 1), and along with trehalose phosphatase (TP), detected by both the XP-CLR and PHS scans (Table S10 in Additional file 3), is involved in trehalose metabolism. The overexpression of TH was shown to increase drought stress tolerance in *Arabidopsis* [68] and trehalose accumulation in transgenic rice expressing TP was associated with an increased tolerance to drought, salt, and cold stresses [69].

The regions subjected to recent selection detected by the PHS scan showed a more substantial overlap in pairwise comparisons between the genomes than the regions detected using the XP-CLR and *F*_ST_ scans (Table S24 in Additional file 1) suggesting that during wheat improvement selection most likely operated on standing variation. However, the presumed adaptive variants with high-PHS in the overlapping homoeologous regions under selection are rarely found in the same line (Figure S19 in Additional file 1). In the vast majority (75%) of overlapping homoeologous regions, no wheat lines in the panel possessed the high-PHS variants simultaneously in two genomes. Although there are known examples where allelic variation at the three wheat homoeologs affect the corresponding traits [70-72], our findings indicate that multiple parallel changes across homoeologous regions have rarely been favored by selection. Rather, it is likely that any favored variant at any one of the homoeologous regions may be sufficient to provide a fitness benefit, thereby expanding the target size of advantageous mutations. The rarity of individual genotypes with positively selected alleles in different homoeologous regions possibly indicate that additional advantageous mutations in other homoeologs either: (1) do not provide fitness benefit; or (2) are absent in a population subjected to improvement selection.

Gene expression studies have demonstrated that, in wheat, the homoeolog-specific transcriptional dominance affects up to 19% of genes [32] with different homoeologs being preponderant in different groups of functionally related genes and showing the tissue- or development-specific patterns of expression [33]. These data are consistent with partitioning of gene function among the duplicated homoelogous genes [34,35], which could also affect the distribution of selective sweeps among the wheat genomes. One possibility is that selection acts on the preferentially expressed homoeolog. For example, most of the natural variation impacting the vernalization requirement in wheat is located in the *VRN-A1* homoeolog [70], which is the homoeolog expressed at the highest level [73].

## Conclusions

The sequence-based diversity map reported here is an important step towards the detailed characterization of DNA sequence polymorphism in the complex allopolyploid genome. The recently developed wheat genomic reference [27] allowed us to catalogue common SNPs and small-scale indel polymorphisms from the low-copy fraction of the genome, describe their patterns of chromosomal distribution and inter-variant association, and identify variants that may have an impact on gene function based on the available annotation. A developed haplotype map will be a valuable tool for imputing genotypes and transferring sequence-level variation data across multiple gene mapping projects, thereby increasing the power and precision of trait mapping in GWAS and helping to understand better the basis of complex phenotypic traits.

Our data helped us gain insights into historic selective events and identify candidate selection targets associated with regions harboring genes controlling important agronomic traits or involved in response to biotic and abiotic stress stimuli. Our results suggest that directional selection in allopolyploids rarely acted on multiple parallel advantageous mutations across homoeologous regions. A favored variant at any one of the homoeologous regions appears to provide sufficient fitness benefit. By broadening the set of targets for selection, allopolyploidy may have played a critical role in the evolution of adaptation in wheat and contributed to wheat’s success as a globally grown crop. Duplicated homoeologs may increase the likelihood of recovering beneficial alleles by expanding the advantageous mutation target size, and/or capturing allelic diversity present in different homoeologous genomes.

## Materials and methods

### Selection of wheat accessions

A total of 62 diverse hexaploid wheat lines (Table S1 and Figure S1 in Additional file 1) were selected to represent the genetic diversity of a large wheat collection that was previously genotyped using the 9 K wheat iSelect assay [13]. In addition, attempts were made to select accessions from major wheat growing areas (Figure 1b). The sample included 26 landraces, 29 cultivars, six breeding lines, and one synthetic wheat (broadly used in the breeding programs of CIMMYT, Mexico), among which 49 and 13 lines show spring and facultative/winter growth habits, respectively. The sample size of 62 lines allows for detection of most common variants present in populations at frequencies ≥1.6%.

### Capture assay design

A wheat exome capture (WEC) assay targeted the 107 Mb of non-redundant low-copy regions in the wheat genome. The capture probes were designed using the low-copy number genome assembly (LGC) of the wheat cultivar Chinese Spring [74]. The LGC had chloroplast, mitochondria, and transposon sequences removed and also contained homoeologous copies of genes collapsed into a single contiguous sequence [29]. The LGC was 3.8 Gb in size. We adopted two strategies to reduce the size over which we could design probes and target exonic regions. First, we used the BLASTN program (e-value <1e^-10^) to identify LGC contiguous sequences that were similar to *Brachypodium* exon sequences. Second, we used the same LGC sequence library (BLASTN e-value <1e^-20^) to identify LGC contiguous sequences that matched a set of non-redundant wheat cDNA and EST sequences [18] and transcriptome assemblies generated by 454 sequencing of nine diverse wheat cultivars [13]. Finally, to remove sequence duplications from the contiguous sequences set, we compared the set against itself using the BLASTN program. Similar sequences were identified (95% identity over 100 bp) and the longest contiguous sequence of a matching pair was retained. This process resulted in a design space of 110 Mb that was used to design a final probe set covering 107 Mb currently available from Roche NimbleGen [75]. The WEC assay was designed by the wheat-barley exome capture consortium that also designed an assay for the enrichment of the barley exome [76].

### DNA extraction and sequence capture

Each accession before DNA isolation was self-pollinated for two generations in the greenhouse. DNA was extracted using the DNeasy Plant Maxi Kit (Qiagen, USA) from a single 3-week-old seedling. One μg of DNA was fragmented with the Covaris S220 to obtain an average fragment length of 300 bp. The NEBNext DNA Library Prep Kit (NEB) for Illumina and Illumina TruSeq (TS) indexed (barcoded) adapters were then used for sample library preparation according to NEB protocol with the following exceptions. The PCR Enrichment step from the NEB was replaced with the Ligation Mediated PCR (LM-PCR). The TS-PCR Oligo1 (5′-AATGATACGGCGACCACCGAGA-3′) and TS-PCR Oligo2 (5′- CAAGCAGAAGACGGCATACGAG-3′) were used in the LM-PCR. The LM-PCR products were purified with the QIAquick PCR Purification Kit (Qiagen) followed by the size selection using Agencourt AMPure XP Beads (Beckman Coulter). The libraries were tested on 2100 Bioanalyzer (Agilent Technologies) and the NanoDrop Spectrophotometer (Thermo Scientific). Only samples with average fragment lengths of 200 to 400 bp, A_260/280_ ratio 1.7 to 2.0, and LM-PCR yield >500 ng were pooled and used for sequence capture. Several levels of DNA sample pooling have been used in our study (Table S1 in Additional file 1). One μg of non-pooled or pooled DNA (for example, 1 μg, 500 ng, 333 ng, 250 ng, or 125 ng of each component DNA sample library were used for 0, 2×, 3×, 4×, or 8× pools, respectively) was used in each of the sequence capture hybridizations. The sequence capture was performed as previously described [77].

### Genotyping by sequencing (GBS)

Complexity-reduced sequencing was performed according to the previously described protocol [25] modified from Poland *et al.* [78]. A pooled library was sequenced on one lane of Illumina HiSeq 2000 (2 × 100 bp paired-end). All subsequent analyses were carried out using the same approaches for exome capture and GBS datasets.

### Selection of alignment parameters for polyploid genome

For mapping reads to the polyploid wheat genome we have developed a three-step iterative alignment strategy with parameters optimized to map reads uniquely to the different wheat subgenomes. Parameters were optimized using a subset of 29,716 Illumina 2 × 100 bp reads generated for cultivar RAC875. These reads map to 100 homoeologous sets of genes (three copies per gene) from the wheat CSS assemblies [27]. We mapped Illumina reads to this reference set of 300 genes using various combinations of Bowtie’s alignment parameters (Figure S4 in Additional file 1).

### Alignment to the CSS assemblies

Raw paired-end Illumina reads were quality filtered using the default setting of NGS QC toolkit v2.3 [79], retaining reads if ≥70% of the bases had a quality score ≥20. Only paired-end reads were mapped to the CSS assemblies using Bowtie1 v.0.12.7 [80] and Bowtie2 v.2.0.0 [81]. We applied the three-step iterative mapping strategy using Bowtie to perform ungapped read alignment (Figures S4 and S5 in Additional file 1). Reads that do not align using more stringent criteria were reused for subsequent rounds of alignment with lower stringency. To find insertion/deletion (indel) polymorphisms, we performed gapped alignment using Bowtie2 v. 2.0.0 [81] with the following parameters: -N 1, -L 75, -D 20, -R 3, --no-mixed, --end-to-end.

More than 4.7 billion paired-end reads were generated from the population of 62 accessions. On average 62% of quality-filtered reads generated by sequence capture were mapped covering more than 321 Mb of the hexaploid wheat genome (Table S2; Figure S3 in Additional file 1). On average 51% of quality-filtered reads generated by the GBS approach were mapped covering more than 247 Mb of mostly intergenic space (Table S3 in Additional file 1).

### Efficiency of homoeologous target capture

The WEC assay included probes covering 107 Mb of non-redundant target space in the wheat genome. To assess the ability of the capture assay to enrich for targets from the three homoeologous wheat genomes, we compared the WEC against the CSS assemblies and retained only those that had three best BLASTN hits (e-value <1 × 10^-10^); one hit per genome. There were 47,739 homoeologous gene sets that fit these criteria. The log_2_ ratio of average coverage depth for each of these gene sets in pair-wise genome comparisons (A vs. B, B vs. D, and A vs. D) was distributed normally with the mean centered at 0, suggesting that homoeologous targets are captured with equal efficiency (Figure 1c).

To assess *in silico* the total size of the regions targeted by the WEC assay in the wheat genome we used the BLAT program [82] to align the WEC design space against the CSS contigs (alignment length >100 bp, similarity >80-99%) (Figure S2 in Additional file 1).

### Capture efficiency

Analysis of alignments generated using the bowtie [81] and BWA [83] aligners showed that 95% of the 107 Mb WEC design space was covered at 30× depth and 99% of the design space was covered by at least one read. Approximately 78% of the annotated high confidence exons in the CSS [27] were covered by at least one read. The average depth of read coverage for annotated exons in each accession was 8.1, 8.6, and 8.4 for the A, B, and D genomes, respectively (Table S4 in Additional file 1), further suggesting no bias in the efficiency of homoeologous genome capture.

### Variant calling and filtering

Each accession’s BAM file was sorted and indexed using Samtools version 0.1.18 [84] for variant calling. Next, GATK version 2.2-8 [85] was used to realign reads around indels. The program Picard v. 1.62 [86] was used to remove duplicate reads in the realigned BAM files. Finally, base quality recalibration was performed using the GATK program [85]. We identified 53.8 million raw variants using the Unified Genotyper from GATK following the GATK instructions for exome capture datasets. Subsequent recalibration of variant quality scores was performed using the default parameters of the VQSR tool in GATK [85]. As ‘true variant’ calls VQSR utilizes genotypes obtained with the wheat 90 K SNP assay [14]. All variants were filtered further to remove sites that had <46 (<75%) accessions genotyped or >2 alleles. We retained sites that had no more than one accession with a heterozygous call, or sites where the heterozygous call was due to a single read of the secondary allele (possibly sequencing error).

Due to the low-coverage depth obtained in the GBS dataset (on average of 1.04 read), genotype calling was performed only for alleles that were present in at least two different accessions in the population. The high level of genotype calling concordance between the WEC and GBS datasets was indicative of the high accuracy of the applied GBS genotype calling approach (see details in section ‘Error rate estimation’).

### Error rate estimation

We assessed the accuracy of genotype calling using four datasets. First, we compared variant calls generated for the cultivar Chinese Spring in our sequence capture experiment with the CSS assemblies that have also been generated using the same wheat cultivar. Of the filtered sites we found that 1,505,400 SNPs had genotype calls for cultivar Chinese Spring and 16,736 of these genotype calls were different from the reference sequence for an error rate of 1.1%. Similarly we estimated an error rate of 1.5% for indels where we found 1,576 indels that disagreed with the 106,438 indels that had a genotype call for Chinese Spring. The rare variant (MAF ≤1.6%) calling accuracy assessed in a set of 12,426 singletons unique to cultivar Chinese Spring depended on the read coverage depth (Figure S7 in Additional file 1). By applying a ≥10 read coverage cutoff we obtained a singleton error rate of 4.6% for SNPs and 3.4% for indels.

Second, to exclude the possibility that the assessed error rates are not impacted by the quality of CSS reference assembly, we compared our variant calls generated for Chinese Spring with the published Chinese Spring BAC sequences generated using the Sanger approach [87]. We found 260 discrepancies out of 85,973 SNPs for an error rate of 0.3%.

Third, we estimated the concordance of our genotype calls with the genotype calls generated using the 90 K iSelect genotyping assay [14]. We selected only those SNP assays from the 90 K assay that were polymorphic in our population and whose flanking sequences could be unambiguously mapped to a single location in the CSS assemblies. Out of 27,147 homozygous genotypes called for these SNPs in our alignments, 520 were different from the SNP assay dataset, for an overall concordance rate of 98.1%.

Fourth, we have assessed the concordance of genotype calls by comparing the WEC and GBS datasets including the shared 10,028 SNP and 838 indel sites (Figure 1d). The level of genotype calling concordance between these two datasets was 97.2% for SNPs and 95.4% for indels.

### SNP annotation

The impact of SNPs on coding sequences was assessed with the SNPEffect program [88] using the 124,201 high confidence gene models predicted in the CSS contigs. We found that the proportion of SNPs identified in the intergenic regions is higher than that assigned to the coding regions. This pattern of SNP distribution is likely defined by several factors. First, WEC is designed using the low-copy fraction of wheat genome that overlapped with the sequences of wheat transcripts. These sequences do not always overlap with the high-confidence gene annotations of the CSS contigs used for annotating SNPs. It is possible that with better annotation of the wheat genome, new genes corresponding to these low-copy genomic regions could be found in WEC design. Second, the WEC co-captures regions outside the target region including introns and non-coding DNA. These regions are overall more genetically diverse and can also contribute to SNPs in the intergenic space.

Functional annotation and enrichment were determined using BLAST2GO software [89]. The ancestral state at SNP sites was inferred by comparing the SNP-flanking sequences among the A, B, and D genomes and with sequences of diploid wheat ancestors *T. urartu* [90] and *Ae. tauschii* [45]. We were able to infer the ancestral allelic states for 754,080 of the 1.57 million SNPs.

### Genetic diversity analyses

Due to variation in the depth of read coverage across the genome genotype calling rate for rare variants present only once in the population can vary from region to region. To reduce the effect of false negative rare variant calls on the local estimates of diversity, all analyses were performed using the variants with MAF >1.6%.

Population structure was inferred using PCA and Structure analyses [91]. Structure was run 10 times using the admixture model with correlated allele frequencies for 20,000 burn-in 100,000 MCMC iterations. Results of independent runs were summarized using CLUMPP [92]. The optimal number of populations (K) in the dataset assessed by plotting the probability of data ln Pr (X|K) for each value of K was K = 4. The proportion of each accession’s ancestry in one of the K populations is presented on Figure 1b as a pie chart.

Recently, it was demonstrated that the estimates of commonly used diversity statistics (*F*_ST_, Tajima’s *D*, and *π*) obtained using restriction enzyme-based sequencing can deviate from true values [93]. Ascertainment bias in GBS data can result from the mutations at the restriction enzyme cut sites, sensitivity of the PstI enzyme to DNA methylation, or a high proportion of missing genotypes in low-coverage re-sequencing datasets. Allele frequency estimates obtained for the same sites in the GBS and WEC datasets showed good correlation (Figure S20 in Additional file 1). However, we observe a slight decrease in correlation values with the increase of the proportion of missing data in the GBS dataset. To reduce the potential effect of missing data on our analyses this dataset was excluded from the estimates of diversity statistics.

The order of the CSS contigs along the wheat chromosomes was established using the combination of ‘genome zipper’ [94] and a high-density genetic map developed by the population sequencing (PopSeq) of 90 recombinant inbred lines [27]. The approximate physical positions of ordered CSS contigs on the chromosomes were inferred using a framework created by cross-linking high-density SNP wheat genetic map [14], wheat deletion bin map [95], and barley genome sequence [96]. Comparison of the inferred physical positions with the positions of CSS contigs on the published sequence of the chromosome 3B pseudomolecule [28] showed a high level of correlation (Pearson correlation *r*^2^ = 0.97). The ordered CSS contigs were used to perform sliding window analyses. *π* and Tajima’s *D* estimates for 2 Mb sliding windows (1 Mb step) with >10 kb of CSS contigs covered by reads were calculated using LDHat (v. 2.2) [97]. Outliers of *π* and Tajima’s *D* were defined by the 2.5 percentiles of the test statistic distribution. *F*_ST_ was calculated by SNP using the R package adegenet (cran.us.r-project.org) by contrasting cultivars and landraces. A sliding window analysis (2 Mb with a 1 Mb step) was used to generate average estimates of *F*_ST_. Outliers were calculated as the 97.5 percentile of all window values within the genome. Adjacent outlier windows were merged. The thresholds for the cultivar-landrace comparison were 0.158 for the A genome, 0.108 for the B genome, and 0.0925 for the D genome.

For estimating recombination rate we selected 10,517 CSS contigs that contained at least 20 non-singleton SNPs. The historical recombination parameter (ρ = 4Ne) was estimated using a composite likelihood approach implemented in the Maxhap program [47]. Regions of the wheat genome showing elevated levels of historic recombination were defined as those falling above 97.5 percentile of the genome-wide ρ distribution. The critical values of ρ for each genome were 7.5 × 10^-3^, 8.5 × 10^-3^/kb, and 9.7 × 10^-3^/kb for the A, B, and D genomes, respectively.

Pair-wise estimates of LD were obtained for SNPs with a MAF ≥0.05 by measuring *r*^2^ as previously described [13]. The average length of pair-wise shared haplotypes in the population was calculated around each SNP according to the previously described procedure [98]. The genetic map distances were taken from the wheat SNP map developed using the 90 K SNP assay [14].

### Distribution of PFAM domains in the CSS contigs

The nucleotide sequences of the CSS gene models for *T. aestivum* were mapped against the wheat survey sequence using GMAP [99]. Output was filtered for hits with ≥98% identity and ≥80% coverage. PFAM domains were identified in the amino acid sequences of the CSS gene models using PFAM hidden Markov models (release 26) from the Sanger Institute [100] and HMMER (version 3.0b3) from the Howard Hughes Medical Institute Janelia Farm Research Campus [101]. The PFAM searches were performed using the default parameters and the results were filtered for hits with ≥90% coverage.

### Phylogenetic tree construction

A total of 20,000 SNPs were randomly selected from the dataset to estimate pairwise distances among the 62 accessions using the R package ‘ape’ V3.0.6 [102]. The bootstrap values for each node of the tree are the average of 10 separate bootstrap runs, each comprising 100 iterations. In total, 45 nodes of 60 have at least a 70% chance of being grouped into the same cluster, and 56 had at least a 50% chance of being grouped together. The neighbor-joining tree is shown on Figure S1B in Additional file 1.

### Distribution of *Ae. tauschii* (genotype AL8/78) alleles across the D genome chromosomes

To determine which of the D genome alleles corresponds to the AL8/78 genotype, 100 bp flanking sequences of each D genome SNP were extracted and compared against the genomic sequence of *Ae. tauschii* AL8/78 genotype [45]. In total, we could determine AL8/78 alleles for 105,294 SNPs. The distribution of AL8/78 alleles along the wheat chromosomes was estimated in the population of the 26 landraces. The average frequency of the AL8/78 allele in a sliding window of 3 Mbp was plotted along the wheat chromosomes.

### Detection of selective sweeps

The PHS statistic was calculated as described by Toomajian *et al*. [53]. Utilizing this statistic we have higher likelihood of detecting genomic regions harboring alleles present at intermediate frequencies in the population [53] than those alleles that have reached high frequency. Thresholds of the PHS statistic were determined by taking the 97.5 percentile of the distribution of PHS values for each SNP within different allele frequency classes (in a window of 0.05). For detecting selective sweeps the wheat genome was split into 50-kb windows; each window was assigned a maximum PHS value for SNPs in the window. Neighboring windows located within 1 Mb that contained outlier SNPs were merged. Annotated genes harboring the outlier SNPs were used for PFAM domain enrichment analyses. Enrichment analysis of GWAS SNPs included sites with a minor allele frequency >3% and a significance level *P* <10^-3^.

The selection scan using the XP-CLR approach is robust to assumptions regarding recombination rates and demography [52] and compares the allele frequency differentiation and the extent of linked variation between two populations (cultivars vs. landraces) to detect regions where change in frequency occurred too quickly to be caused by random drift. The XP-CLR scan was run with the grid size of 50 kb, the window size of 1 cM, and the maximum number of SNPs fixed at 500. The critical values for putative selection targets were estimated based on the 97.5 percentile of the test statistic distribution for each wheat genome. Since our population includes both spring and winter wheat lines, one of the concerns was that population differentiation between these growth habit groups can be mistaken for the signals of selection. However, we believe that at the genome-wide level, inclusion of spring and winter wheat lines should not have a large effect on the detected signals of selection because the level of genetic differentiation between the spring and winter wheat in our population was shown to be very low (mean genome-wide *F*_ST_ = 0.03). This low *F*_ST_ was observed previously [13] and attributed to the common practice to use lines from both growth habit groups in the same breeding programs, as well as to the heterogeneity of the genetic basis of flowering time regulation in wheat where the same phenotypic outcome can be obtained by mutations at several independent genetic loci. In addition, we have identified regions of the wheat genome showing extreme genetic differentiation (*F*_ST_) or XP-CLR test statistics between spring and winter wheat. Out of 372 XP-CLR outliers in the landrace-cultivar comparison only nine (2.4%) partially overlapped with the outliers in the spring-winter wheat comparison (shown in Table S18 in Additional file 10). Out of 168 outliers in the *F*_ST_ scan of cultivars and landraces only six (3.6%) overlapped with the *F*_ST_ outliers in the spring-winter wheat comparison. No overlap of genetically differentiated genomic regions between spring and winter wheat with the outliers of the genetic diversity scan was found. None of the genes located in the genomic regions that showed the signature of selection in at least two scans reported in Table S10 (Additional file 3) fall within the genomic regions differentiated between spring and winter wheat. Overall, these analyses suggest that the genetic differentiation between spring and winter wheat in our sample should not have significant impact on the results of our selection scans.

To test the significance of the observed overlap between the selective sweeps located on the homoeologous chromosomes, we have randomly permuted 10,000 times a genome-wide set of 50-kb windows. The proportions of windows that overlapped among each pairwise comparison of the wheat genomes was ranked and compared to observed data to calculate empirical *P* value.

### Comparison of selective sweeps with previously characterized marker-trait associations

To check if the putative selective sweeps identified in our scans harbored loci associated with agronomic traits, we analyzed published marker-trait associations detected using the DArT markers [103], and 9 K and 90 K iSelect SNP assays [13,14]. The DArT markers were mapped to the CSS contigs using the BLAT program (best hit with minimum alignment length >150 bp). Out of 57 DArT markers that could be mapped to the ordered CSS contigs, 37 markers fell into the regions detected in the PHS scan, and one fell into a region detected by both the PHS and *F*_ST_ scans (Table S21 in Additional file 11). Out of 555 SNP markers mapped to the ordered CSS contigs, 422 mapped to the regions identified in the PHS scan. We found five SNPs in the XP-CLR regions, three of which overlapped with the PHS regions. One SNP marker was located within the region showing reduced level of diversity. There were 30 SNP-trait associations that fell into high *F*_ST_ regions, of which 18 overlapped with the high-PHS regions.

### Imputation

Genotype imputation was performed using Beagle v.4 [104] with the following parameters: ‘window = 5,000 overlap = 500 burns-its = 10 impute-its = 10’. To increase the accuracy of imputation, the settings of burns-its and impute-its have been increased from the default settings (burns-its = 5, impute-its = 5) to 10 (according to recommendations in user’s manual). The accuracy of genotype imputation assessed in windows including from 1,000 to 5,000 markers for cultivars Avalon and Rialto showed no significant differences (Figure S21 in Additional file 1). A setting of window = 5,000 was selected because of its computational efficiency.

To test the accuracy of imputation, we have sequentially selected each cultivar from the panel of 62 lines and masked all variants, except approximately 14,000 SNPs overlapping between the WEC and 90 K SNP iSelect array. At these SNP sites at least 75% of accessions in both datasets had genotype calls. The remaining 61 cultivars were used as a reference panel for imputing 649,502 SNPs that were ordered along the wheat chromosomes. After imputation, genotypes were filtered using different thresholds of genotype probability assessed by Beagle. The filtered predicted genotypes in each cultivar were compared with the actual genotype calls obtained by WEC sequencing to assess the accuracy of imputation. Relationships between the genotype probability threshold, proportion of missing data after filtering, and imputation accuracy are presented on Figure 3a and Table S12 in Additional file 4. The number of imputed genotypes varied among chromosomes depending on the number of polymorphic SNPs from the 90 K iSelect assay on each chromosome. Because of the low level of polymorphism, a relatively low number of SNPs could be imputed in the wheat D genome. However, due to its high LD levels, imputation accuracy on the D genome chromosomes was higher than that in the A and B genomes.

A similar imputation strategy was used for imputing genotypes in the panel of 678 wheat lines used for GWAS of disease resistance.

### Genome-wide association study (GWAS)

#### Plant materials

A total of 838 bread wheat accessions from the Arthur Watkins Collection were obtained from the Australian Winter Cereals Collection, Tamworth. This collection includes a large number of phenotypically diverse wheat landraces collected from 32 countries in the 1920s to 1930s [105]. The 838 accessions were grown under field conditions and single plant selections made for 678 accessions on the basis of plant type, rust resistance, and maturity. Despite some maturity differences, all 678 land race accessions flowered by the end of October. Seed from the purified 678 accessions can be obtained from the Plant Breeding Institute, Cobbitty, upon request (contact urmil.bansal@sydney.edu.au).

#### Phenotyping

Following seed bulk up for the single plant selections, the purified 678 accessions were grown under field conditions during 2006 and 2007 at the Lansdowne site of the University of Sydney. Each accession was grown as a single 1 m row. Field trials were artificially inoculated with *Puccinia striiformis* f. sp. *tritici* (Pst) pathotype 134 E16A+; *P. triticina* (Pt) pathotypes 104-1,(2),3,(6),(7),11 + Lr37; 104-1,(2),3,(6),(7),11,13; 104-1,(2),3,(6),(7),9,11; 76-3,5,9,10 + Lr37; 10-1,3,(7),9,10,11,12 and *P. graminis* f. sp. *tritici* (Pgt) pathotypes 98-1,2,3,5,6 and 34-1,2,7 + Sr38. The pathotype designations are provided according to McIntosh *et al.* [106]. These pathotypes carry partial virulence for the genes given in the parenthesis. Stripe rust, leaf rust, and stem rust responses were recorded using a 1–9 scale, where 1 was highly resistant and 9 was highly susceptible [107]. For stripe rust disease, two records were taken within each year. The consistency of phenotypic evaluations across years was tested by calculating the Pearson correlation coefficient. For leaf, stripe, and stem rust phenotyping datasets the correlation coefficients were 0.75, 0.71-0.87, and 0.57, respectively.

#### Genotyping

The 678 landrace accessions were genotyped using the Infinium iSelect 90 K SNP assay, the content of which is reported to have minimal ascertainment bias for the analysis of diverse wheat landraces [14]. Genotyping was performed on the iScan instrument according to the manufacturer’s protocols (Illumina). SNP genotype calling was performed using GenomeStudio v2011.1 software (Illumina) and the genotype calling algorithm reported in Wang *et al.* [14]. Monomorphic markers and SNPs with more than 10% missing data (due to the presence of null alleles or poor genotype call rates) were removed. The genetic map developed by genotyping multiple mapping populations with the 90 K array was anchored to the GenomeZipper and PopSeq maps by comparing the sequences of 90 K array SNPs with the sequences of wheat chromosome assemblies [27].

#### Association mapping

Mixed model variance component analysis of mean phenotypic values was performed using the R package GAPIT [108]. The information about the relationship among accessions in the population was provided as a kinship matrix (random effect). The effect of population structure was controlled by using the first three principal components from the principal component analysis (fixed effect). The *P* value <1 × 10^-3^ used to filter markers was selected based on the previous studies of agronomic traits in wheat and barley demonstrating that this threshold provides adequate accuracy for detecting marker-trait associations, as was validated independently in bi-parental mapping populations [50,63,109]. In a follow-up analysis we successfully validated five GWAS regions by comparing with previously published studies, or by mapping a trait in a bi-parental mapping population in our study (shown on Figure 3b and in Table S14 in Additional file 6).

### Validation of GWAS signals in mapping populations

#### Plant materials

Mapping in the populations of recombinant inbred lines (RIL) was used to validate several marker-trait associations identified in the GWAS including two characterized and one previously uncharacterized disease resistance loci. The population segregating for leaf rust resistance gene *Lr67* included 124 F_3_/F_4_ lines derived from a cross between Thatcher and RL6077 [110]. The population segregating for stripe rust resistance gene *Yr51* was comprised of 89 F_6_ RILs derived from a single heterozygous F3 plant #5515 from a cross between Watkins’ line PBI769 and Westonia [111]. The population segregating for a new stem rust resistance gene on chromosome 7A was comprised of 96 F_6_ RILs derived from a cross between Watkins’ line PBI562 and Yitpi (henceforth, PBI562/Yitpi).

#### Identification of SNPs linked to rust genes

SNPs associated with rust resistance genes segregating in each mapping population were identified using bulk segregant analysis (BSA) [112] and selective genotyping (SG).

For BSA, resistant and susceptible bulks were prepared by pooling equal amounts of genomic DNA from at least 20 plants for each phenotypic class. An artificial F1 sample was prepared by combining an equal amount of DNA from each of the two bulks. The bulked DNA samples, artificial F1, and parental lines were genotyped using a custom Infinium iSelect bead chip assay on the iScan instrument following the manufacturer’s instructions (Illumina Ltd.). The Thatcher/RL6077 and PBI No. 769/Westonia were genotyped using the 9 K and 90 K iSelect genotyping arrays, respectively. The SNPs were assessed for putative linkage by comparing the normalized theta values for each sample as described in Hyten *et al.* [113]. Polymorphism was considered to be linked to a rust resistance gene when the normalized theta values for the resistant bulk and resistant parent, and susceptible bulk and susceptible parent were similar, and when the normalized theta value for the artificial F_1_ samples was about halfway between that of the other samples.

For SG, at least 15 resistant and 15 susceptible plants from PBI No. 562/Yitpi cross and its parents were genotyped using the 90 K iSelect bead chip assay. Polymorphism was considered to be linked to a rust resistant gene when the majority (>90%) of individuals within each phenotypic class were fixed for the expected parental allele.

#### Validation of GWAS-linked SNPs using bi-parental mapping crosses

The genomic location of SNPs associated with rust disease resistance in the GWAS analysis were compared with those linked to mapped rust resistance genes in each of the bi-parental mapping crosses. GWAS-associated SNPs were considered to co-locate with the mapped resistance genes when the linked SNPs in each study were located at (or very near) the same position in the 90 K consensus SNP genetic map [14]. In instances where the linked markers in either study were not present in the 90 K consensus SNP map, the GWAS-associated SNPs were considered to co-locate with the mapped resistance genes when the CSS contigs tagged by the SNPs [14] co-located in the PopSeq map [27]. The co-location of SNPs associated with rust disease resistance in the GWAS analysis with mapped rust resistance genes in the bi-parental mapping populations is shown in Figure 3b and Table S14 in Additional file 6.

### Data availability

The sequencing data have been deposited in the NCBI Short Read Archive under accession number SRP032974. Datasets used for diversity analyses, genotype imputation, and GWAS are available from the project website [114]. Variant calling datasets in the VCF format for both WEC and GBS are available from the USDA GrainGenes and URGI websites [115,116].

## Additional files

Additional file 1:
**Tables S1-S7, S9, S11, S19-S20, S22-S24** **and** **Figures S1-S21. Table S1.** List of wheat lines sequenced. **Table S2.** Summary of exome capture results. **Table S3.** GBS of the wheat diversity panel. **Table S4.** Mean depth of read coverage. **Table S5.** Distribution of wheat exome capture and GBS variants among the genomic features. **Table S6.** Loss-of-function variants. **Table S7.** Distribution of indels among genomes and their effect on codon reading frame. **Table S9.** Quantile distribution of diversity statistics. **Table S11.** Inter-genomic correlations of diversity statistics. **Table S19.** Genomic regions showing the evidence of selection. **Table S20.** Percentiles of test statistic distribution around domestication and local adaptation genes. **Table S22.** Overlap of selective sweeps identified using different methods. **Table S23.** Distribution of over-represented PFAM domains. **Table S24.** Proportion of overlapping selective sweeps between the wheat genomes. **Figure S1.** PCA and NJ tree of the wheat diversity panel. **Figure S2.** Wheat genome sequences targeted by the WEC assay. **Figure S3.** Summary of read mapping. **Figure S4.** Selection of alignment parameters for bowtie. **Figure S5.** Flowchart of data processing. **Figure S6.** Variant distribution among various genomic features. **Figure S7.** Estimation of varinat calling error rates for singletons. **Figure S8.** Distribution of indel sizes. **Figure S9.** Enrichment of different functional classes of variants over synonymous variants. **Figure S10 - S15.** Diversity distribution along the wheat chromosomes. **Figure S16.** Genetic differentiation between cultivars and landraces. **Figure S17.** GWAS using imputed and non-imputed datasets. **Figure S18.** Manhattan plot of the XP-CLR statistics. **Figure S19.** The proportion of wheat lines in our sample that have the high-PHS variants in the both genomes of the overlapping homoeologous regions. **Figure S20.** Estimation of reference allele frequency in the GBS and WEC datasets. **Figure S21.** Impact of window size variation on genotype imputation accuracy. Additional file 2: Table S8. The distribution of non-synonymous and synonymous variants among different genes classified according to GO terms and PFAM domains. Additional file 3: Table S10. Gene models located in the regions detected by two selection scans (PHS/ XP-CLR, FST/PHS or FST/XP-CLR). Additional file 4: Table S12. The impact of genotyping probability cutoff on imputation accuracy and proportion of missing data. The table shows the number of correctly imputed genotypes/number of genotypes after filtering using a given genotype probability threshold (proportion of missing in the filtered dataset in %). Additional file 5: Table S13. The results of disease-resistance phenotyping performed for GWAS. Additional file 6: Table S14. The results of GWAS of disease resistance in wheat. Additional file 7: Table S15. The results of validation of three GWAS SNPs by mapping in the populations of recombinant inbred lines. Additional file 8: Table S16. The comparison of marker-trait associations around the locus on chromosome 7A conferring resistance to stem rust pathogen. Additional file 9: Table S17. The targets of selection identified in the PHS scan. Additional file 10: Table S18. The targets of selection identified using the XP-CLR scan. Additional file 11: Table S21. The overlap of selective sweep regions with published marker-trait associations detected for major agronomic traits in wheat.
