# Supplementary material for: A haplotype map of allohexaploid wheat reveals distinct patterns of selection on homoeologous genomes
Source: Genome Biol. 2015 Feb 26;16(1):48. doi: 10.1186/s13059-015-0606-4 (PMC4389885; doi:10.1186/s13059-015-0606-4)
Supplement: Additional file 1: — Tables S1-S7, S9, S11, S19-S20, S22-S24 and Figures S1-S21. Table S1. List of wheat lines sequenced. Table S2. Summary of exome capture results. Table S3. GBS of the wheat diversity panel. Table S4. Mean depth of read coverage. Table S5. Distribution of wheat exome capture and GBS variants among the genomic features. Table S6. Loss-of-function variants. Table S7. Distribution of indels among genomes and their effect on codon reading frame. Table S9. Quantile distribution of diversity statistics. Table S11. Inter-genomic correlations of diversity statistics. Table S19. Genomic regions showing the evidence of selection. Table S20. Percentiles of test statistic distribution around domestication and local adaptation genes. Table S22. Overlap of selective sweeps identified using different methods. Table S23. Distribution of over-represented PFAM domains. Table S24. Proportion of overlapping selective sweeps between the wheat genomes. Figure S1. PCA and NJ tree of the wheat diversity panel. Figure S2. Wheat genome sequences targeted by the WEC assay. Figure S3. Summary of read mapping. Figure S4. Selection of alignment parameters for bowtie. Figure S5. Flowchart of data processing. Figure S6. Variant distribution among various genomic features. Figure S7. Estimation of varinat calling error rates for singletons. Figure S8. Distribution of indel sizes. Figure S9. Enrichment of different functional classes of variants over synonymous variants. Figure S10 - S15. Diversity distribution along the wheat chromosomes. Figure S16. Genetic differentiation between cultivars and landraces. Figure S17. GWAS using imputed and non-imputed datasets. Figure S18. Manhattan plot of the XP-CLR statistics. Figure S19. The proportion of wheat lines in our sample that have the high-PHS variants in the both genomes of the overlapping homoeologous regions. Figure S20. Estimation of reference allele frequency in the GBS and WEC datasets. Figure S21. Impact of window size variation on genotype impu [file 13059_2015_606_MOESM1_ESM.doc]

**Additional File 1**

A haplotype map of allohexaploid wheat reveals distinct patterns of selection on homoeologous genomes

Katherine W. Jordan, Shichen Wang, Yanni Lun, Laura-Jayne Gardine, Ron MacLachlan, Pierre Hucl, Krysta Wiebe, Debbie Wong, Kerrie Forrest, IWGS Consortium, Andrew G. Sharpe, Christine H. D. Sidebottom, Neil Hall, Christopher Toomajian, Timothy Close, Jorge Dubcovsky, Alina Akhunova, Luther Talbert, Urmil Bansal, Harbans Bariana, Matthew Hayden, Curtis Pozniak, Jeffrey A. Jeddeloh, Anthony Hall, Eduard Akhunov

**This file includes:**

Tables S1-S7, S9, S11, S19-S20, S22-S24

Figures S1-S21

Supplementary Tables

Table S1.

List of wheat lines sequenced.

| **Wheat lines** | **Origin** | **Improvement status** | **Growth habit** | **Region** | **Large region** |
| --- | --- | --- | --- | --- | --- |
| RAC875 | Australia | Cultivar | Spring | Australia | Australia |
| Opata | Mexico | Cultivar | Spring | North and Central America | The Americas |
| W7984 | Mexico | Synthetic | Spring | North and Central America | The Americas |
| PBW343 | India | Cultivar | Spring | South-central Asia | Asia |
| Clear White | USA | Cultivar | Spring | North and Central America | The Americas |
| Vorobey | Mexico | Cultivar | Spring | North and Central America | The Americas |
| Klein Chamaco | Argentina | Cultivar | Spring | South America | The Americas |
| Pavon | Mexico | Cultivar | Spring | North and Central America | The Americas |
| acc2 | USA | Breeding line | Spring | North and Central America | The Americas |
| acc3 | USA | Breeding line | Spring | North and Central America | The Americas |
| acc4 | USA | Breeding line | Spring | North and Central America | The Americas |
| acc1 | USA | Breeding line | Spring | North and Central America | The Americas |
| acc5 | USA | Breeding line | Spring | North and Central America | The Americas |
| PI366716 | Afghanistan | Landrace | Spring | South-central Asia | Asia |
| PI406517 | Nepal | Landrace | Spring | South-central Asia | Asia |
| PI349512 | Switzerland | Landrace | Spring | Western and Northern Europe | Europe |
| PI481923 | Sudan | Landrace | Spring | Northern Africa | Africa |
| PI481718 | Bhutan | Landrace | Spring | South-central Asia | Asia |
| PI382150 | Japan | Landrace | Spring | Eastern Asia | Asia |
| PI366905 | Afghanistan | Landrace | Spring | South-central Asia | Asia |
| PI470817 | Algeria | Landrace | Spring | Northern Africa | Africa |
| PI445736 | Nepal | Landrace | Spring | South-central Asia | Asia |
| Hidhab | Algeria | Cultivar | Spring | Northern Africa | Africa |
| PI477870 | Peru | Landrace | Spring | South America | The Americas |
| Dharwar Dry | India | Cultivar | Spring | South-central Asia | Asia |
| Cham 6 | Syria/Lebanon | Cultivar | Spring | Western Asia | Asia |
| Chakwal 86 | Pakistan | Cultivar | Spring | South-central Asia | Asia |
| Berkut | Mexico | Cultivar | Spring | North and Central America | The Americas |
| PI262611 | Turkmenistan | Landrace | Spring | South-central Asia | Asia |
| PI222669 | Iran | Landrace | Spring | Western Asia | Asia |
| PI278297 | Greece | Landrace | Spring | Southern Europe | Europe |
| PI210945 | Cyprus | Landrace | Spring | Western Asia | Asia |
| PI192569 | Sweden | Landrace | Spring | Western and Northern Europe | Europe |
| PI192147 | Ethiopia | Landrace | Spring | South and East Africa | Africa |
| PI8813 | Iraq | Landrace | Spring | Western Asia | Asia |
| PI565213 | Bolivia | Landrace | Spring | South America | The Americas |
| PI82469 | North Korea | Landrace | Spring | Eastern Asia | Asia |
| PI185715 | Portugal | Landrace | Spring | Southern Europe | Europe |
| PI245368 | Guatemala | Landrace | Spring | North and Central America | The Americas |
| PI166333 | Turkey | Landrace | Spring | Western Asia | Asia |
| PI166180 | India | Landrace | Spring | South-central Asia | The Americas |
| PI177943 | Turkey | Landrace | Spring | Western Asia | Asia |
| PI192001 | Angola | Landrace | Spring | South and East Africa | Africa |
| PI153785 | Brazil | landrace | spring | South America | The Americas |
| Marquis | Canada | Cultivar | Spring | North and Central America | The Americas |
| Neepawa | Canada | Cultivar | Spring | North and Central America | The Americas |
| AC Barrie | Canada | Cultivar | Spring | North and Central America | The Americas |
| Chinese Spring | China | Cultivar | Spring | Eastern Asia | Asia |
| Utmost | Canada | Cultivar | Spring | North and Central America | The Americas |
| Rialto | United Kingdom | Cultivar | Winter | Western and Northern Europe | Europe |
| Truman | USA | Cultivar | Winter | North and Central America | The Americas |
| 49-2914 H1096 | Argentina | Breeding line | Facultative | South America | The Americas |
| 102 | Chile | Cultivar | Facultative | South America | The Americas |
| 93 | Bulgaria | Cultivar | Facultative | Western and Northern Europe | Europe |
| Estacao | Portugal | Cultivar | Winter | Southern Europe | Europe |
| Taxi | United Kingdom | cultivar | Winter | Western and Northern Europe | Europe |
| PR267 | United States | cultivar | Winter | North and Central America | The Americas |
| Alabasskaja | Kazakhstan | cultivar | Winter |  | Asia |
| Roemer Winter | Germany | cultivar | Winter | Western and Northern Europe | Europe |
| 407-IV/60 | Bosnia and Herzegovina | cultivar | Facultative | Western and Northern Europe | Europe |
| 403 | Chile | cultivar | Winter | South America | The Americas |
| Avalon | United Kingdom | Cultivar | Winter | Western and Northern Europe | Europe |

Table S2.

Summary of exome capture read alignment results.

| **Wheat accession** | **Raw PE reads** | **Quality filtered reads (PE)** | **Aligned Bowtie** | **Aligned Bowtie2** | **Combined aligned** | **Percent aligned^a^** | **Average depth of coverage^b^** |
| --- | --- | --- | --- | --- | --- | --- | --- |
| RAC875 | 42394327 | 36997595 | 19481848 | 2934047 | 22415895 | 0.61 | 14.01 |
| Opata | 38184767 | 33379269 | 18456243 | 2417129 | 20873372 | 0.63 | 13.05 |
| W7984 | 38337100 | 33698056 | 17451706 | 2881910 | 20333616 | 0.60 | 12.71 |
| Pbw 343 | 39859916 | 34990261 | 18451349 | 2714057 | 21165406 | 0.60 | 13.23 |
| Clear White | 45807051 | 42624167 | 24416066 | 2841857 | 27257923 | 0.64 | 17.04 |
| Vorobey | 53342151 | 49668784 | 27986750 | 3509700 | 31496450 | 0.63 | 19.69 |
| Klein Chamaco | 48696130 | 45365836 | 26137250 | 2968254 | 29105504 | 0.64 | 18.19 |
| Pavon | 47200899 | 43938255 | 25276196 | 2884011 | 28160207 | 0.64 | 17.60 |
| acc2^c^ | 47729984 | 43632063 | 23827048 | 3063442 | 26890490 | 0.62 | 16.81 |
| acc3^c^ | 47693513 | 43316225 | 24143285 | 2945347 | 27088632 | 0.63 | 16.93 |
| acc4^c^ | 49508485 | 45120172 | 24845051 | 3139765 | 27984816 | 0.62 | 17.49 |
| acc1^c^ | 55523763 | 50806637 | 27956640 | 3486566 | 31443206 | 0.62 | 19.65 |
| acc5^c^ | 58500054 | 51360997 | 26305966 | 3636491 | 29942457 | 0.58 | 18.71 |
| PI366716 | 59213506 | 52040930 | 27378398 | 3431096 | 30809494 | 0.59 | 19.26 |
| PI406517 | 52040878 | 45665108 | 24243147 | 2937388 | 27180535 | 0.60 | 16.99 |
| PI349512 | 64752923 | 56910966 | 28737515 | 3947972 | 32685487 | 0.57 | 20.43 |
| PI481923 | 64706971 | 57110322 | 29718863 | 3849314 | 33568177 | 0.59 | 20.98 |
| PI481718 | 47113912 | 41305669 | 22849668 | 2454636 | 25304304 | 0.61 | 15.82 |
| PI382150 | 53474777 | 47043596 | 24413269 | 3272966 | 27686235 | 0.59 | 17.30 |
| PI366905 | 50515482 | 44338962 | 23383094 | 2917965 | 26301059 | 0.59 | 16.44 |
| PI470817 | 50364459 | 45729847 | 26148461 | 2958609 | 29107070 | 0.64 | 18.19 |
| PI445736 | 48955203 | 44205394 | 25876652 | 2585056 | 28461708 | 0.64 | 17.79 |
| Hidhab | 50435769 | 45968764 | 26775469 | 2794182 | 29569651 | 0.64 | 18.48 |
| PI477870 | 48034524 | 43511635 | 24844693 | 2837851 | 27682544 | 0.64 | 17.30 |
| Dharwar Dry | 61910522 | 55916432 | 32592712 | 3466399 | 36059111 | 0.64 | 22.54 |
| Cham 6 | 51775361 | 46760481 | 26927238 | 2963003 | 29890241 | 0.64 | 18.68 |
| Chakwat 86 | 49686510 | 44777793 | 25844082 | 2770937 | 28615019 | 0.64 | 17.88 |
| Berkut | 57142201 | 51704203 | 29823990 | 3304590 | 33128580 | 0.64 | 20.71 |
| PI262611 | 56604880 | 53568639 | 27952383 | 3952343 | 31904726 | 0.60 | 19.94 |
| PI222669 | 60287352 | 57171532 | 24992074 | 8109742 | 33101816 | 0.58 | 20.69 |
| PI278297 | 33911131 | 32150419 | 15401510 | 3551064 | 18952574 | 0.59 | 11.85 |
| PI210945 | 44746892 | 42438097 | 17974306 | 6050121 | 24024427 | 0.57 | 15.02 |
| PI192569 | 14749386 | 13976758 | 7032112 | 1368381 | 8400493 | 0.60 | 5.25 |
| PI192147 | 11214927 | 10595104 | 5204544 | 1122784 | 6327328 | 0.60 | 3.95 |
| PI8813 | 11141212 | 10487589 | 5613522 | 741612 | 6355134 | 0.61 | 3.97 |
| PI565213 | 46264819 | 43346752 | 23500950 | 2853136 | 26354086 | 0.61 | 16.47 |
| PI82469 | 40383842 | 38021698 | 20859757 | 2767234 | 23626991 | 0.62 | 14.77 |
| PI185715 | 28725900 | 27108780 | 14827135 | 1892577 | 16719712 | 0.62 | 10.45 |
| PI245368 | 30549499 | 28754508 | 16196155 | 1915459 | 18111614 | 0.63 | 11.32 |
| PI166333 | 40941834 | 38494744 | 21487099 | 2591570 | 24078669 | 0.63 | 15.05 |
| PI166180 | 42935463 | 40522870 | 20801002 | 4034720 | 24835722 | 0.61 | 15.52 |
| PI177943 | 48371719 | 45522812 | 23047358 | 4832268 | 27879626 | 0.61 | 17.42 |
| PI192001 | 36816718 | 34827454 | 19620096 | 2266276 | 21886372 | 0.63 | 13.68 |
| PI153785 | 36347818 | 34279135 | 19078324 | 2336058 | 21414382 | 0.62 | 13.38 |
| Marquis | 79502533 | 74943964 | 47953646 | 4010730 | 51964376 | 0.69 | 32.48 |
| Neepawa | 68429245 | 61958971 | 37757475 | 3417224 | 41174699 | 0.66 | 25.73 |
| AC Barrie | 70612736 | 63738538 | 38666520 | 3650715 | 42317235 | 0.66 | 26.45 |
| Chinese Spring | 430435679 | 389304426 | 248765087 | 11381420 | 260146507 | 0.67 | 162.59 |
| Rialto | 421492430 | 384823424 | 232100129 | 16407004 | 248507133 | 0.65 | 155.32 |
| Truman | 422657241 | 389369664 | 250902358 | 15074334 | 265976692 | 0.68 | 166.24 |
| Utmost | 424986104 | 388841570 | 249151005 | 16127157 | 265278162 | 0.68 | 165.80 |
| H1096 | 45864730 | 42248535 | 22993861 | 3945398 | 26939259 | 0.64 | 16.84 |
| 93 | 53893474 | 48425965 | 24981211 | 4999522 | 29980733 | 0.62 | 18.74 |
| 102 | 117099751 | 107888562 | 55148352 | 10871678 | 66020030 | 0.61 | 41.26 |
| Estacao | 47715379 | 43300070 | 22447562 | 5202811 | 27650373 | 0.64 | 17.28 |
| Taxi | 53813746 | 50005858 | 27967180 | 3975776 | 31942956 | 0.64 | 19.96 |
| PR267 | 42282512 | 39150009 | 22247914 | 3051254 | 25299168 | 0.65 | 15.81 |
| Alabasskaja | 50534370 | 46183563 | 26804585 | 3499875 | 30304460 | 0.66 | 18.94 |
| Roemer Winter | 58806551 | 53681668 | 31350994 | 3966325 | 35317319 | 0.66 | 22.07 |
| 407-IV/60 | 53697959 | 49559493 | 28997588 | 3617493 | 32615081 | 0.66 | 20.38 |
| 403 | 47689731 | 43301369 | 25138033 | 3296041 | 28434074 | 0.66 | 17.77 |
| Avalon | 76130146 | 76085247 | 35345040 | 6177435 | 41522475 | 0.55 | 25.95 |

^a^The combined aligned number of paired end reads of total amount of quality filtered paired end reads.

^b^Average depth of coverage per diploid genome.

^c^Breeding lines from California, USA.

Table S3.

Genotyping by sequencing of the wheat diversity panel.

| **Accession** | **Raw reads (PE)** | **QC passed reads (PE)** | **Aligned reads^a^** | **Percent aligned^b^** |
| --- | --- | --- | --- | --- |
| AC Barrie | 338107 | 310682 | 143866 | 0.46 |
| acc1 | 4490582 | 4320078 | 2232212 | 0.52 |
| acc2 | 2551703 | 2454201 | 1235298 | 0.50 |
| acc3 | 3309237 | 3154440 | 1679281 | 0.53 |
| acc4 | 2349112 | 2255943 | 1216856 | 0.54 |
| acc5 | 3994456 | 3841884 | 1998397 | 0.52 |
| Berkut | 3217534 | 3080546 | 1499082 | 0.49 |
| Chakwal 86 | 3928728 | 3740664 | 1864339 | 0.50 |
| Cham 6 | 2065852 | 1972956 | 1016031 | 0.51 |
| Clear White | 5202086 | 4982213 | 2610962 | 0.52 |
| Chinese Spring | 2128240 | 2059207 | 1095678 | 0.53 |
| Dharwar Dry | 3580683 | 3411694 | 1719371 | 0.50 |
| Hidhab | 2392230 | 2289956 | 1189986 | 0.52 |
| Klein Chamaco | 3466675 | 3318444 | 1696131 | 0.51 |
| W7984 | 3395139 | 2804577 | 1427271 | 0.51 |
| Marquis | 1847730 | 968210 | 474000 | 0.49 |
| Neepawa | 796245 | 764771 | 314279 | 0.41 |
| Opata | 2240492 | 2153848 | 1161802 | 0.54 |
| Pavon | 3111223 | 3020247 | 1583385 | 0.52 |
| Pbw 343 | 2882532 | 2882532 | 1218245 | 0.42 |
| PI153785 | 3846538 | 3677292 | 1890563 | 0.51 |
| PI166180 | 2142968 | 2074784 | 1103274 | 0.53 |
| PI166333 | 2408745 | 2313816 | 1186459 | 0.51 |
| PI177943 | 2427649 | 2353947 | 1262017 | 0.54 |
| PI185715 | 3144328 | 2856649 | 1454418 | 0.51 |
| PI192001 | 3164788 | 3039512 | 1543853 | 0.51 |
| PI192147 | 3059079 | 2924759 | 1454900 | 0.50 |
| PI192569 | 2492671 | 2378175 | 1257291 | 0.53 |
| PI210945 | 2289622 | 2198015 | 1141069 | 0.52 |
| PI222669 | 2323100 | 2233138 | 1179909 | 0.53 |
| PI245368 | 2327442 | 2224003 | 1142066 | 0.51 |
| PI262611 | 2996538 | 2874797 | 1493886 | 0.52 |
| PI278297 | 2643927 | 2522071 | 1262162 | 0.50 |
| PI349512 | 2960366 | 2860653 | 1471003 | 0.51 |
| PI366716 | 4004530 | 3903190 | 2013708 | 0.52 |
| PI366905 | 3623321 | 3467758 | 1771087 | 0.51 |
| PI382150 | 3538254 | 3393208 | 1698591 | 0.50 |
| PI406517 | 3063548 | 2941587 | 1540409 | 0.52 |
| PI445736 | 2482990 | 2393044 | 1171340 | 0.49 |
| PI470817 | 2538218 | 2129055 | 1091014 | 0.51 |
| PI477870 | 3468438 | 3328007 | 1701950 | 0.51 |
| PI481718 | 1300578 | 1257351 | 650220 | 0.52 |
| PI481923 | 2988295 | 2862957 | 1480688 | 0.52 |
| PI565213 | 3181733 | 3034985 | 1613479 | 0.53 |
| PI82469 | 2128443 | 2031311 | 1039937 | 0.51 |
| PI8813 | 2252069 | 2174886 | 1135499 | 0.52 |
| RAC 875 | 3712732 | 3555493 | 1817032 | 0.51 |
| Utmost | 1261039 | 1194746 | 594493 | 0.50 |
| Vorobey | 7317495 | 7020701 | 3509050 | 0.50 |
| 93 | 2034222 | 917097 | 520848 | 0.57 |
| 102 | 781998 | 335544 | 192407 | 0.57 |
| Estacao | 1475824 | 641881 | 363391 | 0.57 |
| Taxi | 1165665 | 579147 | 313956 | 0.54 |
| PR267 | 1990026 | 962885 | 521607 | 0.54 |
| Alabasskaja | 2055154 | 1034081 | 554329 | 0.54 |
| Roemer Winter | 2027448 | 1033024 | 565040 | 0.55 |
| 407-IV/60 | 218719 | 108624 | 59304 | 0.55 |
| 403 | 2150027 | 1086586 | 606259 | 0.56 |
| Total | 156277113 | 141705852 | 72744980 | 0.51 |

^a^Aligned reads contain both gapped and ungapped alignment results.

^b^The combined aligned number of paired end reads of total amount of quality filtered paired end reads.

Table S4.

Mean depth of coverage (per diploid genome equivalent) by chromosome and genome for exons, genes, and 1 kb up- and downstream of the annotated genes for all accessions.

| **Chromosome** | **All accessions** | | |
| --- | --- | --- | --- |
|  | **Exons** | **Genes** | **1 k Up/Down** |
| 1 | 7.90 | 6.64 | 2.22 |
| 2 | 8.32 | 6.96 | 2.30 |
| 3 | 9.30 | 7.77 | 2.72 |
| 4 | 7.90 | 6.64 | 2.22 |
| 5 | 8.40 | 6.93 | 2.28 |
| 6 | 8.68 | 7.27 | 2.37 |
| 7 | 8.85 | 7.34 | 2.46 |
| **Genome** |  |  |  |
| A | 8.45 | 7.06 | 2.39 |
| B | 8.09 | 6.74 | 2.24 |
| D | 8.62 | 7.18 | 2.43 |
| **Total** | 8.39 | 6.99 | 2.35 |
|  |  |  |  |

Table S5.

Distribution of wheat exome capture (WEC) and genotyping-by-sequencing (GBS) variants among the genomic features.

| **Approach** | **SNP class** | **SNP subclass** | **Total** | **A gen.** | | **B gen.** | **D gen.** |
| --- | --- | --- | --- | --- | --- | --- | --- |
| **WEC** | All SNPs |  | 1341350 | 490348 | | 645406 | 205596 |
|  | Non-syn. | Total | 77920 | 27645 | | 37194 | 13081 |
|  |  | PTC | 1508 | 548 | | 710 | 250 |
|  |  | Read through | 39 | 14 | | 19 | 6 |
|  |  | Start lost | 159 | 71 | | 66 | 22 |
|  |  | Start gained | 2481 | 890 | | 1190 | 401 |
|  | Syn. |  | 70490 | 23423 | | 35262 | 11805 |
|  | Intronic |  | 166741 | 58791 | | 82545 | 25405 |
|  | 3’ UTR |  | 29265 | 10797 | | 13720 | 4748 |
|  | 5’ UTR |  | 11440 | 3956 | | 5556 | 1928 |
|  | Upstream^a^ |  | 108992 | 39955 | | 53229 | 15808 |
|  | Downstream^a^ |  | 120873 | 46005 | | 56602 | 18266 |
|  | Intergenic |  | 755629 | 279776 | | 361298 | 114555 |
|  | Indels | Total | 147064 | 53986 | | 68104 | 24974 |
|  |  | CDS | 10112 | 3569 | | 4561 | 1982 |
|  |  | UTR | 15013 | 5417 | | 7103 | 2493 |
|  |  | Intronic | 32122 | 11388 | | 15362 | 5372 |
|  |  | Intergenic | 89817 | 33612 | | 41078 | 15127 |
| **GBS** | All SNPs |  | 225304 | | 99217 | 73395 | 52692 |
|  | Non-syn. | Total | 5702 | | 1990 | 2158 | 1554 |
|  |  | PTC | 92 | | 37 | 36 | 19 |
|  |  | Read through | 2 | | 1 | 1 | 0 |
|  |  | Start lost | 9 | | 1 | 6 | 2 |
|  |  | Start gained | 202 | | 79 | 90 | 33 |
|  | Syn. |  | 5871 | | 2083 | 2158 | 1630 |
|  | Intronic |  | 13955 | | 5121 | 5138 | 3696 |
|  | 3’ UTR |  | 2495 | | 933 | 944 | 618 |
|  | 5’ UTR |  | 1158 | | 387 | 523 | 248 |
|  | Upstream^a^ |  | 11707 | | 4206 | 4808 | 2693 |
|  | Downstream^a^ |  | 11800 | | 4640 | 4744 | 2416 |
|  | Intergenic |  | 172616 | | 79857 | 52922 | 39837 |
|  | Indels | Total | 14655 | | 5971 | 5066 | 3618 |
|  |  | CDS | 422 | | 147 | 163 | 112 |
|  |  | UTR | 678 | | 243 | 290 | 145 |
|  |  | Intronic | 1481 | | 521 | 553 | 407 |
|  |  | Intergenic | 12074 | | 5060 | 4060 | 2954 |

^a^Up to 5 kb from a gene.

Table S6.

Variants resulting in loss of function (LOF). PTC: premature termination codons; SSD: splice-site disruption.

| **Mutation type** | **Total** | **Derived allele known^a^** | **Derived allele results in LOF** | | | |
| --- | --- | --- | --- | --- | --- | --- |
|  |  |  | **Total (%)** | **A gen.** | **B gen.** | **D gen.** |
| PTC | 1,600 | 1,104 | 1,021 (92) | 356 | 480 | 185 |
| SSD | 1,583 | 874 | 668 (76) | 231 | 322 | 115 |

^a^Derived and ancestral allelic states are identified by comparing SNP harboring sequences with the genomes of diploid ancestors and other grass genomes.

Table S7.

Distribution of indels among genomes and their effect on codon reading frame.

|  | **Indels** | **Indels in coding regions** | **Indels result in frame shift^a^** |
| --- | --- | --- | --- |
| Whole genome | 161,719 | 10,339 | 6,944 |
| A genome | 59,957 | 3,645 | 2,508 |
| B genome | 73,170 | 4,624 | 3,054 |
| D genome | 28,592 | 2,070 | 1,382 |

^a^Only first indel from the 5’ end of a gene is counted.

Table S8 (Additional file 2). Distribution of non-synonymous (Non-syn.) and synonymous (Syn.) variants among different genes classified according to GO terms and PFAM domains. Only those functional categories that contain at least 30 SNPs are shown.

Excel file: Additional file 2.xls

Table S9.

Quantile distribution of average pair-wise diversity (π), Tajima’s measure of site-frequency spectrum (D), and historic recombination rate (ρ).

| **Statistic** | **Genome** | **Min** | **2.5 %** | **Mean** | **97.5 %** | **Max** |
| --- | --- | --- | --- | --- | --- | --- |
| π^a^ | Whole genome | 0 | 4.52 × 10^-5^ | 6.44 × 10^-4^ | 2.33 × 10^-3^ | 6.40 × 10^-3^ |
|  | A | 0 | 8.10 × 10^-5^ | 6.71 × 10^-4^ | 2.05 × 10^-3^ | 3.52 × 10^-3^ |
|  | B | 5.45 × 10^-5^ | 1.76 × 10^-4^ | 1.01 × 10^-3^ | 2.90 × 10^-3^ | 6.40 × 10^-3^ |
|  | D | 0 | 2.95 × 10^-5^ | 2.50 × 10^-4^ | 1.06 × 10^-3^ | 3.13 × 10^-3^ |
| Tajima D^a^ | Whole genome | -1.84 | -1.47 | 0.31 | 2.80 | 4.82 |
|  | A | -1.76 | -1.16 | 0.82 | 3.12 | 4.57 |
|  | B | -1.60 | -1.09 | 0.78 | 2.78 | 4.82 |
|  | D | -1.84 | -1.61 | -0.65 | 2.14 | 4.19 |
| ρ^b^ | Whole genome | 0 | 0 | 1.17 × 10^-3^ | 7.82 × 10^-3^ | 0.89 |
|  | A | 0 | 0 | 1.57 × 10^-3^ | 7.47 × 10^-3^ | 0.50 |
|  | B | 0 | 0 | 1.69 × 10^-2^ | 7.74 × 10^-3^ | 0.89 |
|  | D | 0 | 0 | 2.02 × 10^-3^ | 9.74 × 10^-3^ | 0.22 |

^a^Based on 2 Mb windows that contain at least 10kb of sequence covered by mapped reads.

^b^Based on all CSS contigs that contain at least 20 SNPs.

Table S10 (Additional file 3). Gene models in the regions detected by two selection scan (PHS/ XP-CLR, FST/PHS, or FST/XP-CLR). Gene annotation was performed by comparing with the PFAM database.

Excel file: Additional file 3.xls

**Table S11**. Inter-genomic correlations of diversity statistics between homoeologous chromosomes (2 Mb window size).

| **Chromosome/Genome** | **Diversity (Pi)** | ***P* value** | **Tajima's D** | ***P* value** | **F*_ST_*** | ***P* value** |
| --- | --- | --- | --- | --- | --- | --- |
| 1A-1B | 0.206 | 2.24E-05 | -0.03 | 5.40E-01 | 0.003 | 9.62E-01 |
| 1A-1D | 0.38 | 1.78E-15 | -0.024 | 6.34E-01 | -0.053 | 3.67E-01 |
| 2A-2B | 0.447 | 0.00E+00 | 0.171 | 3.24E-05 | -0.066 | 1.71E-01 |
| 2A-2D | 0.362 | 0.00E+00 | -0.405 | 1.12E-23 | -0.023 | 6.48E-01 |
| 3A-3B | 0.009 | 8.31E-01 | -0.091 | 3.97E-02 | -0.023 | 6.52E-01 |
| 3A-3D | 0.417 | 0.00E+00 | 0.012 | 7.89E-01 | 0.105 | 4.71E-02 |
| 4A-4B | -0.055 | 2.48E-01 | 0.01 | 8.26E-01 | -0.015 | 7.89E-01 |
| 4A-4D | -0.003 | 9.53E-01 | -0.024 | 6.13E-01 | 0.036 | 5.47E-01 |
| 5A-5B | 0.298 | 1.04E-11 | 0.099 | 2.67E-02 | -0.13 | 1.23E-02 |
| 5A-5D | 0.301 | 1.21E-11 | 0.007 | 8.73E-01 | 0.062 | 2.45E-01 |
| 6A-6B | 0.292 | 9.30E-11 | -0.105 | 2.17E-02 | 0.09 | 9.15E-02 |
| 6A-6D | 0.483 | 0.00E+00 | 0.015 | 7.51E-01 | 0.276 | 2.64E-07 |
| 7A-7B | 0.324 | 9.33E-15 | 0.158 | 2.28E-04 | 0.15 | 2.34E-03 |
| 7A-7D | 0.305 | 3.80E-13 | -0.076 | 7.87E-02 | 0.165 | 8.41E-04 |
| 1B-1D | 0.191 | 9.40E-05 | 0.011 | 8.22E-01 | -0.094 | 1.02E-01 |
| 2B-2D | 0.561 | 0.00E+00 | -0.226 | 5.45E-08 | -0.033 | 5.10E-01 |
| 3B-3D | -0.005 | 9.07E-01 | -0.042 | 3.50E-01 | -0.001 | 9.85E-01 |
| 4B-4D | 0.24 | 1.60E-07 | 0.03 | 5.24E-01 | 0.089 | 1.08E-01 |
| 5B-5D | 0.354 | 4.44E-16 | 0.11 | 1.46E-02 | 0.014 | 7.87E-01 |
| 6B-6D | 0.255 | 2.34E-08 | -0.088 | 5.71E-02 | 0.031 | 5.65E-01 |
| 7B-7D | 0.368 | 0.00E+00 | -0.009 | 8.33E-01 | 0.033 | 5.06E-01 |
| A-B | 0.005 | 7.81E-01 | -0.025 | 1.46E-01 | -0.002 | 9.08E-01 |
| A-D | 0.132 | 8.66E-15 | -0.021 | 2.26E-01 | 0.084 | 3.41E-05 |
| B-D | 0.05 | 3.36E-03 | -0.015 | 3.72E-01 | -0.016 | 4.33E-01 |

Table S12 (Additional file 4). Impact of genotyping probability cutoff on imputation accuracy and proportion of missing data. The table shows the number of correctly imputed genotypes / number of genotypes after filtering using a given genotype probability threshold (proportion of missing in the filtered dataset in %).

Excel file: Additional file 4.xls

Table S13 (Additional file 5). Disease-resistance phenotyping for GWAS.

Excel file: Additional file 5.xls

Table S14 (Additional file 6). GWAS of disease resistance phenotypes.

Excel file: Additional file 6.xls

Table S15 (Additional file 7). Validation of three GWAS SNPs by mapping in the populations of recombinant inbred lines.

Excel file: Additional file 7.xls

Table S16 (Additional file 8). Comparison of marker-trait associations around the locus on chromosome 7A conferring resistance to stem rust pathogen. Results are shown on Figure 3B.

Excel file: Additional file 8.xls

Table S17 (Additional file 9). Targets of selection identified using the PHS scan.

Excel file: Additional file 9.xls

Table S18 (Additional file 10). Targets of selection identified using the XP-CLR scan.

Excel file: Additional file 10.xlsx

Table S19.

Genomic regions showing the evidence of selection.

| **Test statistic (percentile of distribution)** | **Regions in the extreme tails (n)** | | | |
| --- | --- | --- | --- | --- |
|  | **A genome** | **B genome** | **D genome** | **Total** |
| π (2.5%) | 46 | 50 | 52 | 148 |
| F_ST_ (2.5%) | 55 | 63 | 50 | 168 |
| PHS (97.5%) | 201 | 296 | 205 | 702 |
| XP-CLR (97.5%) | 155 | 125 | 92 | 372 |

**Table S20.**

Percentile of test statistic distribution around genomic regions overlapping with genes associated with wheat domestication and local adaptation.

| **Genes** | **CSS contig** | **Percentile** | **Statistics** | **Putative function**  **or associated QTL** |
| --- | --- | --- | --- | --- |
| *Ppd-A1* | 5262553_2as | 98% | PHS | Photoperiod sensitivity gene |
| *Ppd-B1* | 2504037_2bs | 94% | PHS |  |
| *Ppd-D1* | 171278_2ds | 95% | PHS |  |
| *Q gene-A* | 1750512_5al | 14% | π | AP2 transcription factor |
| *Q gene-B* | 10920529_5bl | 94% | PHS |  |
| *Q gene-B* | 10920529_5bl | 96% | XP-CLR |  |
| *Q gene-D* | 4605422_5dl | 95% | PHS |  |
| *Tg* | 5170471_2bs | 15% | π | Tenacious glume |
| *Rht-B1* | 4913659_4bs | 97.5% | F_ST_ | Gibberellin insensitive transcription factor |
| *Rht-B1* | 4913659_4bs | 99% | PHS |  |
| *Vrn-A1* | 2805369_5al | 91% | XP-CLR | MADS-box transcription factor |
| *Vrn-A1* | 2805369_5al | 91% | PHS |  |
| *Vrn-B1* | 10800239_5bl | 99% | PHS |  |

Table S21 (Additional file 11). Overlap of selective sweep regions with published marker-trait associations detected for major agronomic traits in wheat.

Excel file: Additional file 11.xlsx

**Table S22.**

The number of overlapping selective sweep regions identified by the pair-wise comparison of the selection scan approaches. The regions were considered overlapping if at least one gene was common between them. In some cases, due to size differences among the selective sweep regions, overlap may include more than one region. For example, in the F_ST_/XP-CLR comparison 61 regions (16% of total 372) showing high CLR overlapped with 55 regions (33% of total 168) that were outliers in the F_ST_ scan.

| **Pair-wise comparison of different methods** | **XP-CLR** | **F_ST_** | **PHS** |
| --- | --- | --- | --- |
| **XP-CLR / PHS** | 47 (13%) | - | 47 (1.5%) |
| **F_ST_ / XP-CLR** | 61 (16%) | 55 (33%) | - |
| **PHS / F_ST_** | - | 28 (17%) | 72 (2.4%) |

**Table S23.**

Distribution of over-represented PFAM domains located within the PHS and XP-CLR selective sweeps compared to the genome-wide distribution.

| **Scan** | **Pfam ID** | **Pfam Name** | ***P* value^a^** | **PFAM description** |
| --- | --- | --- | --- | --- |
| XP-CLR | PF00800.13 | PDT | 7.9 × 10^-10^ | Prephenate dehydratase |
|  | PF01472.15 | PUA | 2.8 × 10^-8^ | PUA domain |
|  | PF01591.13 | 6PF2K | 8.7 × 10^-7^ | 6-phosphofructo-2-kinase |
|  | PF01756.14 | ACOX | 8.3 × 10^-6^ | Acyl-CoA oxidase |
|  | PF02881.14 | SRP54_N | 2.1 × 10^-5^ | SRP54-type protein, helical bundle domain |
|  | PF08718.6 | GLTP | 1.4 × 10^-4^ | Glycolipid transfer protein (GLTP) |
|  | PF12171.3 | zf-C2H2_jaz | 5.9 × 10^-4^ | Zinc-finger double-stranded RNA-binding |
|  | PF08068.7 | DKCLD | 5.9 × 10^-4^ | DKCLD (NUC011) domain |
|  | PF01509.13 | TruB_N | 6.0 × 10^-4^ | TruB family pseudouridylate synthase (N terminal domain) |
|  | PF01145.20 | Band_7 | 1.3 × 10^-3^ | SPFH domain / Band 7 family |
|  | PF00124.14 | Photo_RC | 4.6 × 10^-3^ | Photosynthetic reaction centre protein |
|  | PF06964.7 | Alpha-L-AF_C | 5.0 × 10^-3^ | Alpha-L-arabinofuranosidase C-terminus |
|  | PF09066.5 | B2-adapt-app_C | 5.0 × 10^-3^ | Beta2-adaptin appendage, C-terminal sub-domain |
|  | PF00181.18 | Ribosomal_L2 | 6.8 × 10^-3^ | Ribosomal Proteins L2, RNA binding domain |
|  | PF11969.3 | DcpS_C | 6.9 × 10^-3^ | Scavenger mRNA decapping enzyme C-term binding |
|  | PF03143.12 | GTP_EFTU_D3 | 7.7 × 10^-3^ | Elongation factor Tu C-terminal domain |
|  | PF13326.1 | PSII_Pbs27 | 9.3 × 10^-3^ | Photosystem II Pbs27 |
|  | PF00383.17 | dCMP_cyt_deam_1 | 9.3 × 10^-3^ | Cytidine and deoxycytidylate deaminase zinc-binding region |
|  | PF13398.1 | Peptidase_M50B | 9.3 × 10^-3^ | Peptidase M50B-like |
|  | PF00576.16 | Transthyretin | 9.3 × 10^-3^ | HIUase/Transthyretin family |
|  | PF11221.3 | Med21 | 9.3 × 10^-3^ | Subunit 21 of Mediator complex |
|  | PF09349.5 | OHCU_decarbox | 9.3 × 10^-3^ | OHCU decarboxylase |
|  | PF03587.9 | EMG1 | 9.3 × 10^-3^ | EMG1/NEP1 methyltransferase |
|  | PF04614.7 | Pex19 | 9.3 × 10^-3^ | Pex19 protein family |
|  | PF06644.6 | ATP11 | 9.3 × 10^-3^ | ATP11 protein |
|  | PF01238.16 | PMI_typeI | 9.3 × 10^-3^ | Phosphomannose isomerase type I |
|  | PF01204.13 | Trehalase | 9.3 × 10^-3^ | Trehalase |
| PHS | PF00931.17 | NB-ARC | 1.2 × 10^-6^ | NB-ARC domain |
|  | PF11976.3 | Rad60-SLD | 1.9 × 10^-3^ | Ubiquitin-2 like Rad60 SUMO-like |
|  | PF00097.20 | zf-C3HC4 | 4.0 × 10^-3^ | Zinc finger, C3HC4 type (RING finger) |
|  | PF00240.18 | ubiquitin | 4.0 × 10^-3^ | Ubiquitin family |
|  | PF00707.17 | IF3_C | 4.0 × 10^-3^ | Translation initiation factor IF-3, C-terminal domain |
|  | PF05712.8 | MRG | 4.0 × 10^-3^ | MRG |
|  | PF13923.1 | zf-C3HC4_2 | 4.0 × 10^-3^ | Zinc finger, C3HC4 type (RING finger) |
|  | PF14580.1 | LRR | 4.0 × 10^-3^ | Leucine-rich repeat (LRR) |

^a^χ^2^ test’s adjusted *P* value (FDR <0.05).

Table S24.

Proportion of overlapping regions between the wheat genomes. The selective sweep regions associated with wheat improvement were identified by contrasting landraces and cultivars as outlier windows in the XP-CLR and F_ST_ selection scans. The PHS scan was used to detect regions of recent selective sweeps.

| **Selection scan** | **Pair-wise genome comparisons** | **Proportion of overlapping windows in pair-wise genome comparison^a^** | | |
| --- | --- | --- | --- | --- |
|  |  | **A** | **B** | **D** |
| XP-CLR/F_ST_ | **-/A** | - | 0/26 | 0/26 |
|  | **-/B** | 0/25 | - | 1/25 |
|  | **-/D** | 0/10 | 1/10 | - |
| PHS | **-/A** | - | 124/201 | 105/201 |
|  | **-/B** | 104/296 | - | 113/296 |
|  | **-/D** | 127/205 | 141/205 | - |

^a^In some cases, due to size differences among the selective sweep regions, overlap may include more than one region. For example, in the PHS scan 124 out of 201 selected regions in the A genome overlapped with the selected regions in B genome, whereas 104 out of 296 regions in the B genome overlapped with the selected regions in the A genome.

Supplementary Figures


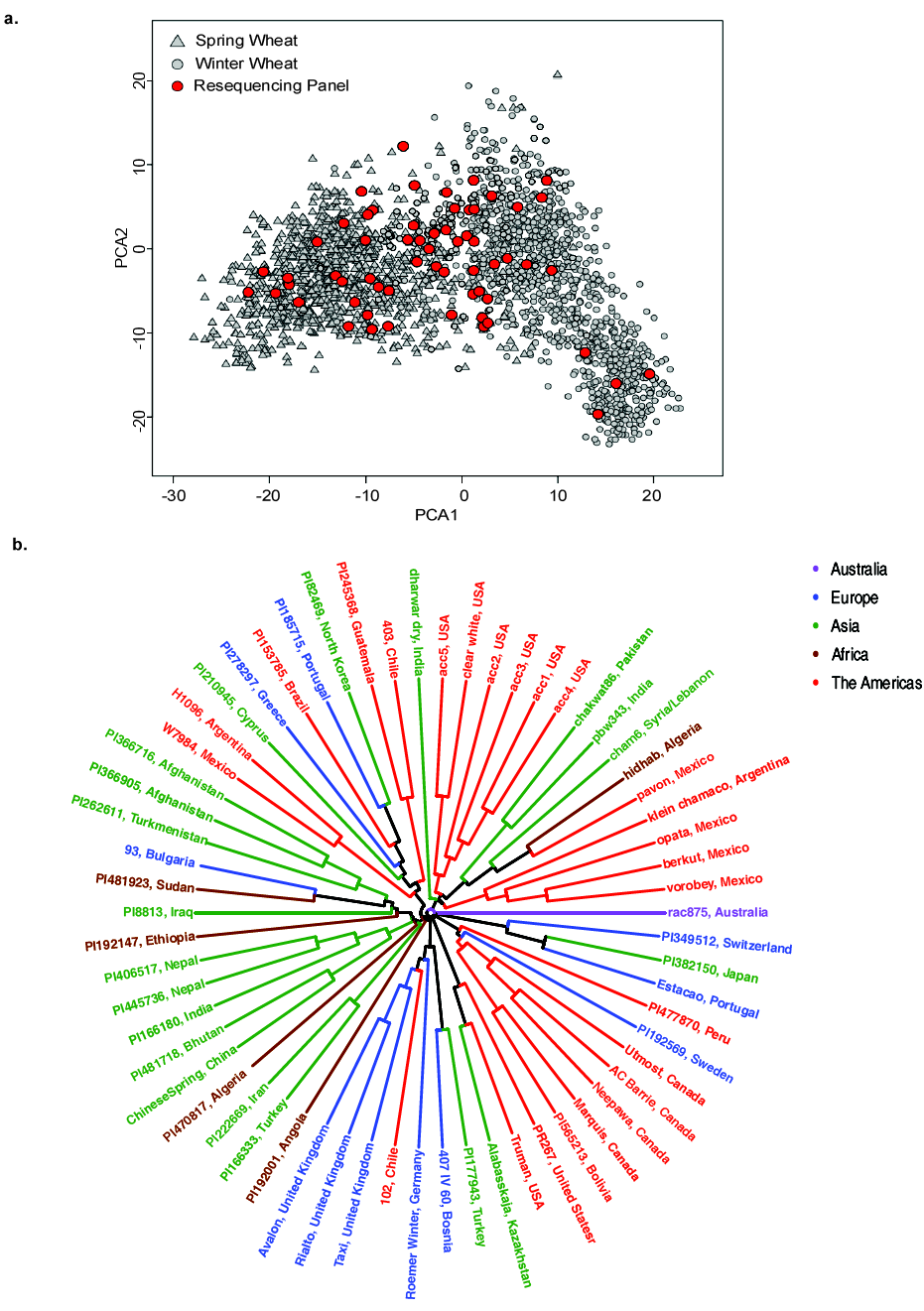


Figure S1.

(**a**) Principal component analysis of the wheat diversity panel. Accessions selected for re-sequencing are shown by red circles; spring and winter wheat accessions are shown by triangles and circles (for details see Supplementary Table 1, Fig. 1B). (**b**) Neighbor joining tree of 62 diverse accessions color coded by continent of origin. Most branches have bootstrap support above 70%. Previous studies demonstrated that the development of regional populations of cultivars occurred through the usage of distinct founding landraces or divergence of populations from the ancestral populations of landraces [13]. Therefore, clustering of landraces and cultivars on the tree does not always coincide geographically. For example, Syrian cultivar Cham 6 was developed using lines derived from the CIMMYT (Mexico) breeding program and, therefore, it clusters closely with the Mexican cultivar Pavon.

Figure S2.

Size of the wheat genome sequence targeted by the WEC assay. (**a**) Increase in the length of the wheat genome sequence aligned to the WEC design space as a function of the similarity level (from 80% to 100% with the step of 1%) used for filtering the alignments generated using the BLAT program. The increase in the length of aligned sequence at any given similarity threshold was expressed as a proportion of sequence length that can be aligned at 1% higher similarity threshold. It appears that the proportion of aligned reads increase significantly when the similarity threshold changed from 99% to 98%. Most likely 98% similarity threshold allows the majority sequences from the homoeologous genomes to align, consistent with the previous estimates of the inter-genomic coding sequence divergence in wheat (2% to 4%). (**b**) Total length of the WEC design sequence (in Mb) aligned to the wheat genome as a function of the similarity threshold used for alignment filtering. With a strict similarity threshold >99%, the total size of the targeted regions (118 Mb) was close to that of the WEC consistent with the selection of only one of the homoeologous copies of each gene for designing the assay. With similarity threshold >95% (similar to inter-genomic divergence in wheat) the total size of targeted regions was 321 Mb, approximately 3× the size of the WEC.

Figure S3.

Mapping of reads to the wheat genome. Bars represent the percent of reads that could be aligned to the CSS assemblies for each of the 62 accessions. Each bar is partitioned into the percent of reads that were mapped uniquely using ungapped (blue) and gapped mapping (maroon), percent reads could not map uniquely (green), and percent of reads that failed the quality filtering step (purple).


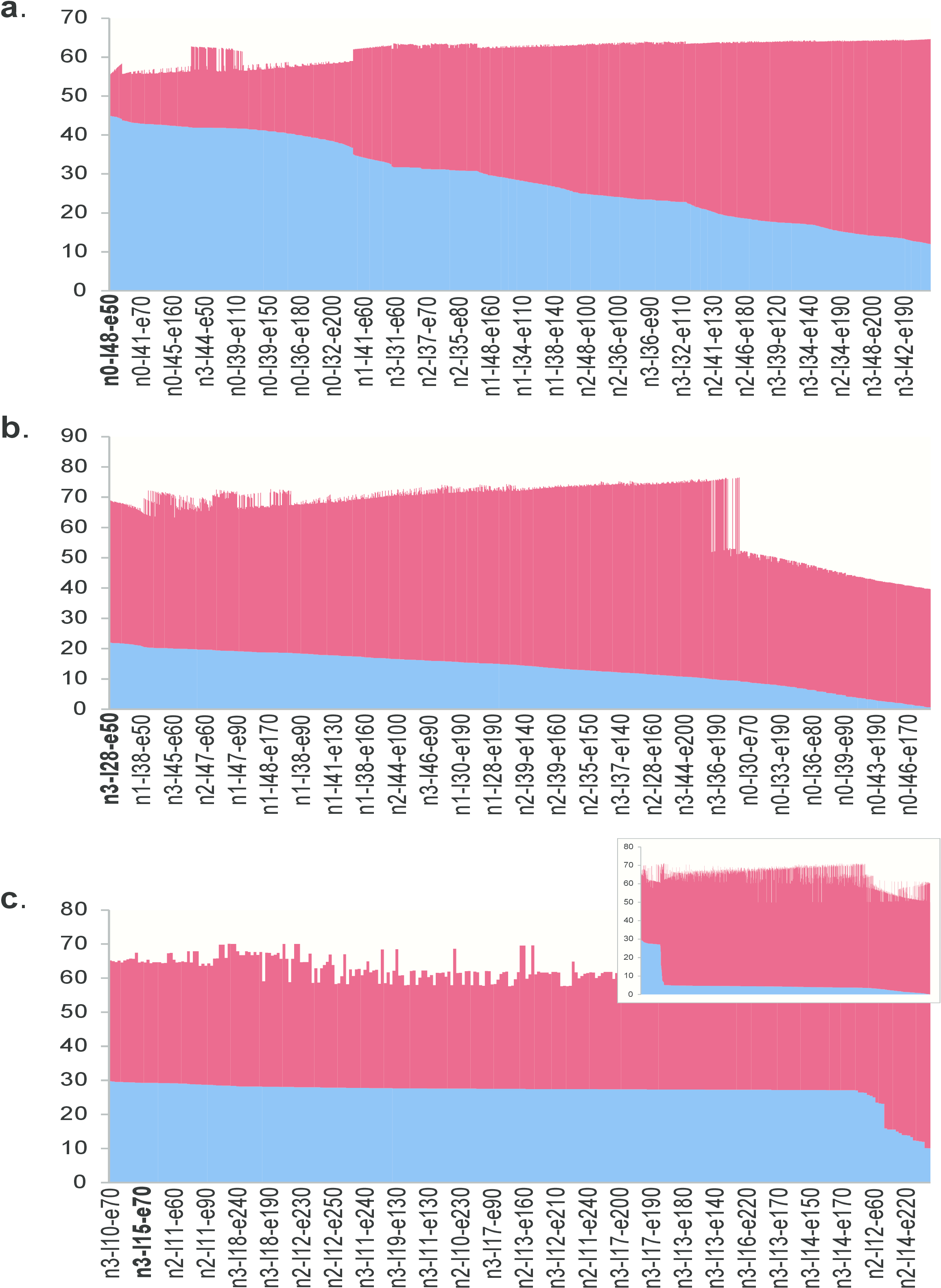


Figure S4.

Selection of alignment parameters for bowtie. Bars represent the percentage of reads that could be aligned to the reference sequence using the set of alignment parameters shown on the x-axis. Each bar is partitioned into the percentage of reads that align uniquely (blue) and have multiple hits (red). All results are sorted by the number of uniquely aligned reads. The insert in box C, shows the entire distribution of values for iteration 3, where the blowup only represents the lower 10% of the distribution that contains the most uniquely mapped reads. Iteration 1 (**a**) contained all reads, iteration 2 (**b**) contained all reads that were not uniquely mapped from the first iteration, and iteration 3 (**c**) contained all reads not uniquely mapped from the first and second iterations. The parameters chosen for subsequent analyses are highlighted in bold.

Figure S5.

Flowchart of data processing. Diagram depicts three stages of data analysis including read processing, read alignment, and variant calling.

Figure S6.

Distribution of SNPs and indels among various genomic features. (**a**) SNPs were classified into the following categories: Synonymous (green), Non-synonymous (orange), UnTranslated Regions (UTR) (yellow), Intron (purple), Upstream and Downstream (maroon, within 5 Kb of a gene), and Intergenic (blue). (**b**) Indels were classified into the following categories: Coding DNA Sequence (CDS) (purple), UTR (green), Intron (maroon), and Intergenic (blue) in total and by genome. (**c**) Distribution of SNPs (blue) and indels (maroon) among the wheat chromosomes.

Figure S7.

Estimation of SNP and indel calling error rates for singleton mutations. For estimating variant calling error rate all singleton mutations unique to cultivar Chinese Spring were compared against the sequences of CSS contigs. The error rate (y-axis) estimate is the percentage of incorrect calls for all singleton mutations at the depth of read coverage equal to or above that specified on the x-axis for SNPs (**a**) and indels (**b**).

Figure S8.

Distribution of insertion/deletion variants in the wheat genome. (**a**) Distribution of insertion/deletion sizes. (**b**) Variation in the length of indels in different genomic features. Blue bars represent the number of indels that have a length that is a multiple of 3; red bars represent number of indels with a length that has a remainder of 1 when divided by 3; and green bars represent indels with a length that has a remainder of 2 when divided by 3. (**c**) The proportion of indels whose length polymorphism is multiple of 3 among different genomic features. A total of 161,719 indels detected in the WEC and GBS datasets were grouped according to their location in coding and non-coding (introns, UTRs, intergenic) regions of the wheat genome. The distribution of indel sizes follows the power law irrespective of their location with most indels being 1 bp long (36% in coding and 55% in non-coding), followed by indels of 2 bp (12% in coding and 14% in non-coding), and 3 bp long (19% in coding and 9% in non-coding). The proportion of indels longer than 3 bp in coding and noncoding regions was 33% and 22%, respectively. We found that out of the 10,339 indels in the coding regions 6,944 (67%) are predicted can result in frame-shift.

Figure S9.

Enrichment of different functional classes of variants over synonymous variants as a function of derived allele frequency in the population. In each derived allele frequency interval (on x-axis) we estimated the proportion of functional variants relative to synonymous variants. Decrease in the level of enrichment for alleles with high derived allele frequency is indicative of purifying selection acting against the detrimental effect of functional mutations. This effect is expressed strongly for alleles resulting in premature termination codons (PTCs). NON-SYN: non-synonymous variants; SDS: splice disrupting sites.

Figure S10.

Diversity along wheat chromosome arms 1A (**a**), 1B (**b**), and 1D (**c**). Average nucleotide diversity π (upper panels) and Tajima’s measure of site frequency spectrum (D, lower panels) was calculated for 2-Mb windows with 1-Mb overlap and plotted along chromosomes 1A (**a**), 1B (**b**), and 1D (**c**). Gray shaded box represents the location of the centromere. Rug plots represent 2.5% (red) and 97.5% outlier windows (blue) for each genome. The genetic differentiation (F_ST_) between wheat cultivars and landraces (2-Mb window, 1-Mb step) is shown by black line. Black X above the plot shows the location of regions of high historic recombination rate (97.5% percentile of ρ distribution).

Figure S11.

Diversity along wheat chromosomes 2A (**a**), 2B (**b**), and 2D (**c**). For details, see Supplementary Fig. 10.

Figure S12.

Diversity along wheat chromosomes 3A (**a**), 3B (**b**), and 3D (**c**). For details, see Supplementary Fig. 10.

Figure S13.

Diversity along wheat chromosomes 4A (**a**), 4B (**b**), and 4D (**c**). For details, see Supplementary Fig. 10.

Figure S14.

Diversity along wheat chromosomes 6A (**a**), 6B (**b**), and 6D (**c**). For details, see Supplementary Fig. 10.

Figure S15.

Diversity along wheat chromosomes 7A (**a**), 7B (**b**), and 7D (**c**). For details, see Supplementary Fig. 10.

**Figure S16.**

The mean level of chromosome arm genetic differentiation (F_ST_) between cultivars and landraces in the A (**a**), B (**b**), and D (**c**) genomes.


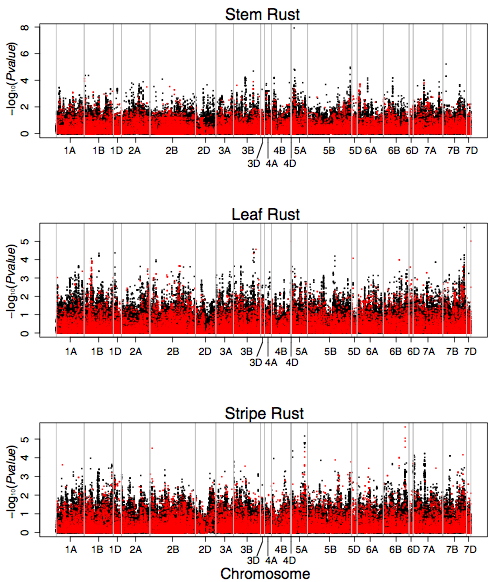


Fig

**Figure S17.**

Genome-wide association mapping in a population of 678 spring wheat lines using imputed and non-imputed datasets. SNP sites directly genotyped using the 90K SNP array are shown as red dots; imputed SNPs are shown as black dots. The number of imputed genotypes varied among chromosomes depending on the number of polymorphic SNPs from the 90K iSelect assay on each chromosome. Because of the low level of polymorphism relatively low number of SNPs could be imputed in the wheat D genome.


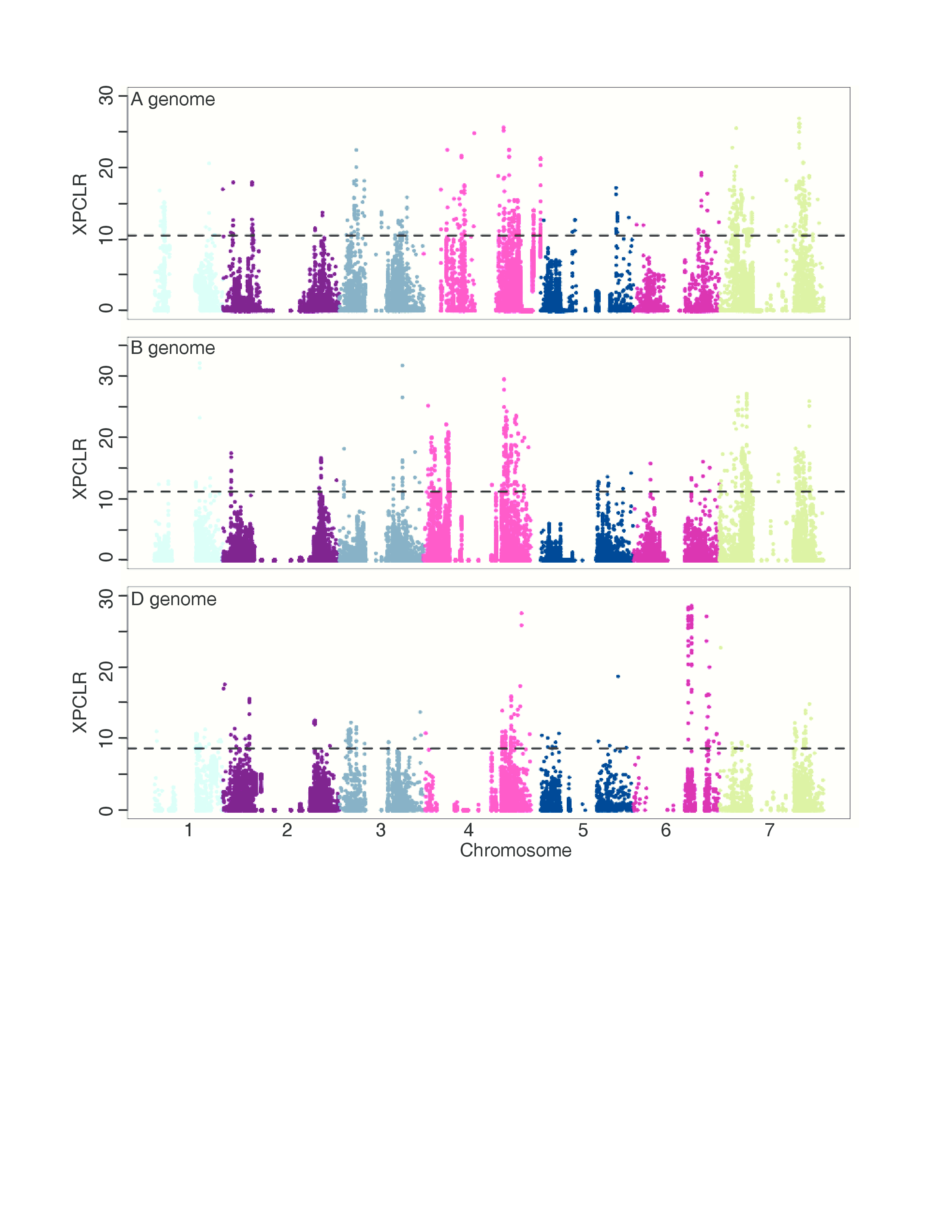


**Figure S18.**

Manhattan plot of the XP-CLR statistics for the A (**top**), B (**middle**), and D (**bottom**) genomes by chromosome. Dashed lines show the 97.5 percentile of the XP-CLR test statistics distribution.

**Figure S19**. The proportion of wheat lines in our sample that have the high-PHS variants in the both genomes of the overlapping homoeologous regions.

Figure S20.

Correlation between the estimates of CSS reference allele frequency in the GBS and WEC datasets. The GBS datasets with 25% (**left**), 50% (**middle**), and 75% (**right**) data-points present included 19,882, 14,887, and 9,155 SNPs, respectively. For each of these three groups we plotted the estimates of CSS reference allele frequency obtained for both the GBS and WEC datasets and fit a regression line (red), as well as included a perfect regression line (black) for comparison.

Figure S21.

The impact of window size variation on genotype imputation accuracy as a function of genotype probability. Five different window sizes including from 1,000 to 5,000 markers were tested for cultivars Avalon and Rialto.
